# Supplementary figures and images for: Growth across life course and cardiovascular risk markers in 18-year-old adolescents: the 1993 Pelotas birth cohort
Source: BMJ Open. 2018 Jan 23;8(1):e019164. doi: 10.1136/bmjopen-2017-019164 (PMC5786082; doi:10.1136/bmjopen-2017-019164)

1993 Pelotas Birth Cohort

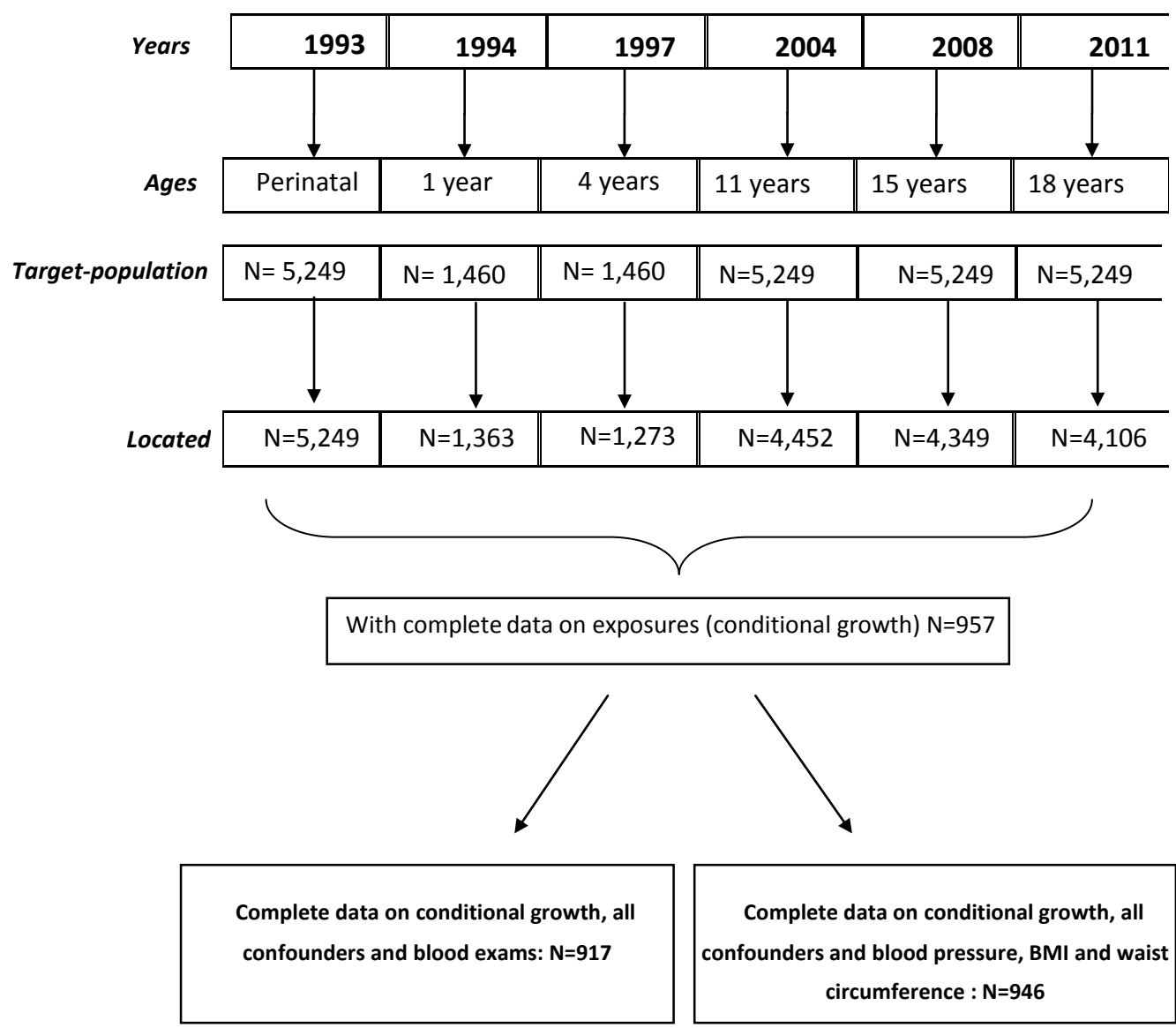

Figure 1. Description of the 1993 Pelotas (Brazil) Birth Cohort.

Supplement: Supplementary file 1 [file bmjopen-2017-019164supp001.pdf]
