# Supplementary material for: Growth across life course and cardiovascular risk markers in 18-year-old adolescents: the 1993 Pelotas birth cohort
Source: BMJ Open. 2018 Jan 23;8(1):e019164. doi: 10.1136/bmjopen-2017-019164 (PMC5786082; doi:10.1136/bmjopen-2017-019164)
Supplement: Supplementary file 2 [file bmjopen-2017-019164supp002.pdf]

Supplementary file 2

Table 1. Mean (SD) for outcomes at 18 years of age in the main analyses samples* and samples comprised by all the adolescents with outcomes measures at the 18 years follow-up, stratified by sex. 1993 Pelotas Birth Cohort.

	Boys					Girls				
**Outcomes**	N	Main analyses sample	N	All participants	p-value*	N	Main analyses sample	N	All participants	p-value*
Plasma glucose (mg/dl)	438	93.60 (20.88)	1933	93.92 (22.65)	0.79	479	88.41 (15.36)	1936	89.74 (18.64)	0.15
HbA1c (%)	438	4.97 (0.60)	1924	4.96 (0.57)	0.74	475	4.86 (0.50)	1910	4.84 (0.52)	0.45
C-reactive Protein (mg/L)	438	0.64 (3.10)	1933	0.67 (3.26)	<0.01	479	1.35 (3.92)	1936	1.35 (3.86)	0.88
Total cholesterol (mg/dl)	438	151.31 (24.46)	1933	152.72 (24.55)	0.27	479	172.29 (30.35)	1936	169.80 (29.19)	0.10
HDL cholesterol (mg/dl)	438	52.78 (8.75)	1933	51.78 (8.75)	0.03	479	59.50 (10.54)	1936	59.84 (10.92)	0.54
LDL cholesterol (mg/dl)	438	83.55 (18.46)	1933	84.28 (20.23)	0.49	479	97.14 (25.42)	1936	93.89 (23.84)	0.01
Triglycerides (mg/dl)	438	70.81 (1.46)	1933	73.56 (1.51)	0.16	479	74.79 (1.44)	1936	74.93 (1.49)	0.62
Systolic blood pressure (mm/Hg)	447	130.3 (11.56)	1979	130.71 (11.90)	0.52	499	115.40 (10.04)	2008	115.06 (9.95)	0.50
Diastolic blood pressure (mm/Hg)	447	70.54 (8.29)	1979	70.95 (7.94)	0.33	499	69.64 (7.83)	2008	69.46 (7.75)	0.64
Body mass index	447	22.99 (4.24)	1970	23.36 (4.23)	0.10	499	23.58 (5.06)	2003	23.52 (4.76)	0.80
Waist circumference (cm)	447	77.51 (9.71)	1972	78.45 (9.61)	0.06	499	73.74 (10.31)	2005	73.75 (9.75)	0.98

Data are arithmetic mean (SD) unless otherwise indicated

* Geometric mean (SD)

*Main analyses samples includes individuals with complete data on all growth measures, all confounders and at least one outcome.

*p-value for T-test. C-reactive protein and triglycerides were log transformed to performed de test.
