# Supplementary material for: Growth across life course and cardiovascular risk markers in 18-year-old adolescents: the 1993 Pelotas birth cohort
Source: BMJ Open. 2018 Jan 23;8(1):e019164. doi: 10.1136/bmjopen-2017-019164 (PMC5786082; doi:10.1136/bmjopen-2017-019164)
Supplement: Supplementary file 3 [file bmjopen-2017-019164supp003.pdf]

Supplementary file 3

Table 1. Differences in mean (SD) for outcomes at 18 years of age between included and excluded participants, stratified by sex. 1993 Pelotas Birth Cohort.

Outcomes	Boys					Girls				
	N	Main analyses sample	N	Excluded	p-value*	N	Main analyses sample	N	Excluded	p-value*
C-reactive Protein (mg/L)	438	0.64 (3.10)	1495	0.68 (3.30)	0.26	479	1.35 (3.92)	1457	1.35 (3.83)	0.95
Total cholesterol (mg/dl)	438	151.31 (24.46)	1495	153.13 (25.11)	0.17	479	172.29 (30.35)	1457	168.9 (28.76)	0.06
HDL cholesterol (mg/dl)	438	52.78 (8.75)	1495	51.70 (8.75)	0.44	479	59.50 (10.54)	1457	59.95 (11.04)	0.43
LDL cholesterol (mg/dl)	438	83.55 (18.46)	1495	84.48 (20.73)	0.40	479	97.14 (25.42)	1457	92.82 (23.20)	<0.00
Triglycerides (mg/dl)	438	70.81 (1.46)	1495	74.38 (1.52)	0.03	479	74.79 (1.44)	1457	74.97 (1.50)	0.91
Systolic blood pressure (mm/Hg)	447	130.30 (11.56)	1532	130.83 (12.00)	0.40	499	115.40 (10.04)	1509	114.99 (9.94)	0.57
Diastolic blood pressure (mm/Hg)	447	70.54 (8.29)	1532	71.09 (7.83)	0.13	499	69.64 (7.83)	1509	69.40 (7.72)	0.56
Body mass index (kg/m^2)	447	22.99 (22.60)	1523	23.47 (23.26)	0.08	499	23.58 (23.14)	1504	23.50 (23.27)	0.75
Waist circumference (cm)	447	77.51 (9.71)	1525	78.72 (78.24)	0.03	499	73.74 (10.31)	1506	73.76 (73.27)	0.97

Data are arithmetic mean (SD) unless otherwise indicated

* Geometric mean (SD)

*Included participants (main analyses samples): includes individuals with complete data on all growth measures, all confounders and at least one outcome.

*p-value for T-test. C-reactive protein and triglycerides were log transformed to performed de test.
