# Supplementary material for: Growth across life course and cardiovascular risk markers in 18-year-old adolescents: the 1993 Pelotas birth cohort
Source: BMJ Open. 2018 Jan 23;8(1):e019164. doi: 10.1136/bmjopen-2017-019164 (PMC5786082; doi:10.1136/bmjopen-2017-019164)
Supplement: Supplementary file 4 [file bmjopen-2017-019164supp004.pdf]

Supplementary file 4

Table 1. Association of conditional relative weight and conditional height with mean arterial pressure at 18 years old in boys and girls. 1993 Pelotas Birth Cohort.

	Mean arterial pressure (MAP)	
	Boys	Girls
Conditional weight		
CWh 0 to1 y	0.04 (-0.04; 0.13)	0.06 (-0.03; 0.14)
CWh 1 to 4 y	0.08 (-0.01; 0.17)	0.14 (0.05; 0.22)
CWh 4 to11 y	0.11 (0.02; 0.19)	0.14 (0.06; 0.22)
CWh 11 to15 y	0.08 (0.01; 0.18)	0.15 (0.07; 0.22)
CWh 15 to 18 y	0.11 (0.02; 0.20)	0.16 (0.09; 0.24)
Conditional length/height		
CH 0 to 1 y	0.12 (0.31; 0.22)	0.05 (-0.03; 0.13)
CH 1 to 4 y	0.07 (-0.20; 0.17)	0.13 (0.04; 0.22)
CH 4 to 11 y	0.20 (0.11; 0.28)	0.17 (0.09; 0.24)
CH 11 to 15 y	0.04 (-0.05; 0.13)	0.05 (-0.02; 0.13)
CH 15 to 18 y	0.05 (-0.04; 0.13)	0.00 (-0.07; 0.07)

CWh: conditional relative weight, CH: conditional height

Data are β (95% CI). The outcome variables were normalized. Regression coefficient (β) values were calculated with linear regression models and indicate the SD change in the outcome per SD change in the predictor. All models were adjusted for mother's education (years of schooling) and household wealth (in minimum wages) at birth and skin color of the adolescent.
