# Supplementary material for: Growth across life course and cardiovascular risk markers in 18-year-old adolescents: the 1993 Pelotas birth cohort
Source: BMJ Open. 2018 Jan 23;8(1):e019164. doi: 10.1136/bmjopen-2017-019164 (PMC5786082; doi:10.1136/bmjopen-2017-019164)
Supplement: Supplementary file 5 [file bmjopen-2017-019164supp005.pdf]

Supplementary file 5

Table 1. Association of weight and length at birth, conditional relative weight and conditional height with cardiovascular risk markers at 18 years old in boys. 1993 Pelotas Birth Cohort.

	Cardiovascular risk markers								
	CRP (mg/L)	TC (mg/dl)	HDL-C (mg/dl)	LDL-C (mg/dl)	TGL (mg/dl)	SBP (mm/Hg)	DBP (mm/Hg)	BMI (kg/m^2)	WC (cm)
Conditional relative weight									
CWh 0 to1 y	0.99	2.41	0.37	1.74	1.01	0.60	0.40	1.19	2.77
CWh 1 to 4 y	1.27	0.83	-0.41	0.85	1.05	1.15	0.39	1.92	4.03
CWh 4 to 11 y	1.24	1.58	-0.84	1.96	1.02	1.17	0.78	2.17	4.37
CWh 11to 15 y	1.14	3.50	0.03	3.10	1.05	0.97	0.60	1.50	2.98
CWh 15 to 18 y	1.18	4.48	-1.16	3.19	1.12	1.00	0.68	2.08	4.73
Conditional length/height									
CH 0 to 1 y	0.99	-0.13	-0.06	0.16	0.99	1.97	0.66	0.49	2.04
CH 1 to 4 y	1.14	1.74	0.29	1.04	1.05	1.25	0.29	0.85	2.43
CH 4 to 11 y	1.05	1.32	-0.88	1.69	1.03	1.80	1.66	0.60	1.84
CH 11 to 15 y	0.96	-0.21	0.33	-0.06	0.97	0.45	0.30	-0.09	0.18
CH 15 to 18 y	0.97	-2.75	-0.36	-1.76	0.97	0.90	0.13	-0.25	0.23

TC: total cholesterol, HDL-C: high-density lipoprotein cholesterol, LDL-C: low-density lipoprotein cholesterol, TGL: triglycerides, DBP: diastolic blood pressure, SBP: systolic blood pressure, BMI: body mass index, CC: waist circumference, CWh: conditional relative weight gain, CH : conditional height gain

Data are β (95% CI). The outcome variables are presented in original scales. Regression coefficient (β) values were calculated with linear regression models and indicate a unit change in the outcome (e.g.: 1 cm or 1 mm/Hg) per SD change in the predictor. All models were adjusted for mother's education (years of schooling) and household wealth (in minimum wages) at birth and skin color of the adolescent. The models for plasma glucose concentrations were further adjusted for time from previous meal.

Table 2. Association of Conditional relative weight and conditional height with cardiovascular risk markers at 18 years old in girls. 1993 Pelotas Birth Cohort

	Cardiovascular risk markers								
	CRP (mg/L)	TC (mg/dl)	HDL-C (mg/dl)	LDL-C (mg/dl)	TGL (mg/dl)	SBP (mm/Hg)	DBP (mm/Hg)	BMI (kg/m^2)	WC (cm)
Conditional relative weight									
CWh 0 to1 y	1.07	0.21	-0.11	-0.40	1.01	0.96	0.26	1.15	2.31
CWh 1 to 4 y	1.24	-1.10	-0.33	-0.98	1.02	1.56	0.96	2.53	4.48
CWh 4 to 11 y	1.21	-1.05	-1.63	0.69	1.00	1.42	1.10	2.77	5.28
CWh 11to 15 y	1.24	0.27	-1.91	1.22	1.05	2.00	0.87	2.02	3.95
CWh 15 to 18 y	1.59	3.88	-1.13	4.49	1.06	2.19	1.02	2.32	4.63
Conditional length/height									
CH 0 to 1 y	0.99	2.36	1.76	0.70	0.99	1.21	0.06	0.10	0.94
CH 1 to 4 y	1.16	1.89	1.04	0.33	1.02	1.53	0.91	0.57	2.20
CH 4 to 11 y	1.01	1.30	0.37	0.90	0.99	1.12	1.65	0.52	1.20
CH 11 to 15 y	0.97	-0.56	-0.75	0.29	0.99	1.12	0.16	-0.03	0.68
CH 15 to 18 y	0.85	0.16	-0.53	0.16	1.02	-0.11	-0.01	-0.23	0.01

HbA1c: glycated hemoglobin, CRP: reactive-C protein, TC: total cholesterol, HDL-C: high-density lipoprotein cholesterol, LDL-C: low-density lipoprotein cholesterol, TGL: triglycerides, DBP: diastolic blood pressure, SBP: systolic blood pressure, BMI: body mass index, CC: waist circumference, CWh: conditional relative weight gain, CH : conditional height gain

Data are β (95% CI). The outcome variables are presented in original scales. Regression coefficient (β) values were calculated with linear regression models and indicate a unit change in the outcome (e.g.: 1 cm or 1 mm/Hg) per SD change in the predictor. All models were adjusted for mother's education (years of schooling) and household wealth (in minimum wages) at birth and skin color of the adolescent. The models for plasma glucose concentrations were further adjusted for time from previous meal.
