# Supplementary material for: Growth across life course and cardiovascular risk markers in 18-year-old adolescents: the 1993 Pelotas birth cohort
Source: BMJ Open. 2018 Jan 23;8(1):e019164. doi: 10.1136/bmjopen-2017-019164 (PMC5786082; doi:10.1136/bmjopen-2017-019164)
Supplement: Author's manuscript [file bmjopen-2017-019164.draft_revisions.pdf]

BMJ Open

BMJ Open is committed to open peer review. As part of this commitment we make the peer review history of every article we publish publicly available.

When an article is published we post the peer reviewers' comments and the authors' responses online. We also post the versions of the paper that were used during peer review. These are the versions that the peer review comments apply to.

The versions of the paper that follow are the versions that were submitted during the peer review process. They are not the versions of record or the final published versions. They should not be cited or distributed as the published version of this manuscript.

BMJ Open is an open access journal and the full, final, typeset and author-corrected version of record of the manuscript is available on our site with no access controls, subscription charges or pay-per-view fees (http://bmjopen.bmj.com).

If you have any questions on BMJ Open's open peer review process please email editorial.bmjopen@bmj.com

BMJ Open

Growth across life course and cardiometabolic risk markers in 18 years old adolescents: the 1993 Pelotas Birth Cohort

Journal:	*BMJ Open*
Manuscript ID	bmjopen-2017-019164
Article Type:	Research
Date Submitted by the Author:	15-Aug-2017
Complete List of Authors:	Buffarini, Romina; Universidade Federal de Pelotas<br>Restrepo-Méndez, Maria Clara; Universidade Federal de Pelotas, Centro Internacional de Equidade<br>Silveira, Vera; Federal University of Pelotas (UFPel), Postgraduate Programme in Epidemiology<br>Goncalves, Helen; Universidade Federal de Pelotas<br>Oliveira, Isabel; Federal University of Pelotas (UFPel), Postgraduate Programme in Epidemiology<br>Menezes, Ana; Federal University of Pelotas (UFPel), Postgraduate Programme in Epidemiology<br>Assuncao, M.C; Federal University of Pelotas, Post-Graduate Program in Epidemiology
**Primary Subject Heading**:	Epidemiology
Secondary Subject Heading:	Public health, Epidemiology
Keywords:	DIABETES & ENDOCRINOLOGY, EPIDEMIOLOGY, PAEDIATRICS, PUBLIC HEALTH

SCHOLARONE™
Manuscripts

Growth across life course and cardiometabolic risk markers in 18 years old adolescents: the 1993 Pelotas Birth Cohort

Romina Buffarini[*1]; María Clara Restrepo-Méndez[2]; Vera Maria Silveira[3]; Helen D. Gonçalves[1], Isabel O. Oliveira[4], Ana Maria Menezes[1], Maria Cecília Formoso Assunção[5]

[1] Post-graduate Program in Epidemiology, Federal University of Pelotas, Marechal Deodoro 1160, 3rd floor, 96020-220, Pelotas, Brazil

[2] International Center for Equity in Health, Federal University of Pelotas, Rua Marechal Deodoro, 1160 3rd floor, 96020-220, Pelotas, Brazil.

[3] Clinical Medical Department, Faculty of Medicine, Federal University of Pelotas, Rua Félix da Cunha 614, Centro, 96010-000, Pelotas, Brazil

[4] Department of Physiology and Pharmacology, Federal University of Pelotas, Campus Universitário s/n, Capão do Leão, 96010-900, Pelotas, Brazil

[5] Department of Nutrition, School of Nutrition, Federal University of Pelotas, Campus Anglo, Gomes Carneiro 1, 96010-900, Pelotas , Brazil

[*]**Corresponding author:** Romina Buffarini. Correspondence address: Marechal Deodoro 1160, 3rd floor, 96020-220, Pelotas, RS, Brasil. Phone/Fax +55 (53) 3284 – 1300. E-mail: romibuffarini@gmail.com.

Manuscript word count: 3148

Abstract word count: 257

For peer review only - http://bmjopen.bmj.com/site/about/guidelines.xhtml

Abstract

Objective: We aimed to evaluate the association between growth trajectories from birth to adolescence and cardiometabolic risk markers levels at age 18 years in a population based cohort. In order to disentangle the effect of weight gain from that of height gain, growth was analyzed using conditional weight relative to linear growth (CWh) and conditional length/height (CH).

Design: Prospective study.

Setting: 1993 Pelotas Birth Cohort, Southern Brazil.

Participants: Individuals that have been followed-up from birth up to adolescence (at birth, 1, 4, 11, 15 and 18 years).

Primary outcome measures: C-reactive protein (CRP), total cholesterol (TC), LDL-C, HDL-C, TGL, systolic and diastolic blood pressure (SBP and DBP), BMI and waist circumference (WC).

Results: In both sexes, greater CWh at 1 year was positively associated with BMI and WC, whereas greater CWh at most age periods in childhood and adolescence predicted increased values of CRP, TC, LDL-C, TGL, SBP, DBP, BMI and WC, and decreased HDL-C. Higher CH during infancy and childhood was positively related with SBP in boys and girls, and with BMI and WC only in boys.

Conclusion: Our study showed that rapid weight gain from 1 year old onwards is positively associated with several markers of cardiometabolic risk at 18 years. The lack of anthropometric data at two years is a limitation in our study, since there is evidence suggesting that the hazards of rapid weight gain appear particularly after the two first years of life. Overall, our study support the "first 1000 days initiative" suggesting that prevention of excessive weight gain after age two years might be important in reducing subsequent cardiometabolic risk.

Key-words: conditional growth, relative weight gain, linear growth, adolescents, cardiometabolic risk, cohort studies.

For peer review only

For peer review only - http://bmjopen.bmj.com/site/about/guidelines.xhtml

Strengths and limitations:

- Population based sample birth cohort. Data collected prospectively over 18 years since birth with high follow ups rates.
- Availability of several anthropometric and biological markers of cardiometabolic risk
- Use of conditional growth to examine highly correlated measurements, and assessment of the separate contributions of linear growth and relative weight gain.
- Use of sub-sample in one of the follow-ups. Multiple hypothesis testing lead to increased Type I error.
- Lack of anthropometric data at two years is a limitation in our study, since there is evidence suggesting that the hazards of rapid weight gain appear particularly after the two first years of life.

Funding: This article is based on data from the study "Pelotas Birth Cohort, 1993" conducted by Postgraduate Program in Epidemiology at Universidade Federal de Pelotas with the collaboration of the Brazilian Public Health Association (ABRASCO). From 2004 to 2013, the Wellcome Trust supported the 1993 birth cohort study. The European Union, National Support Program for Centers of Excellence (PRONEX), the Brazilian National Research Council (CNPq), and the Brazilian Ministry of Health supported previous phases of the study.

Competing interests: None declared

For peer review only

Introduction

Metabolic and cardiovascular diseases (CVDs) are important public health problems, responsible for high morbidity and mortality burden in most regions of the world and causing enormous costs in terms of human and economic resources [1].

Growth trajectories throughout life course, including the fetal period, may have effects on cardiometabolic risk profile later in life [2]. It is well established in the literature that low birthweight (a marker of fetal growth restriction) is a risk factor for type 2 diabetes [3], insulin resistance [4] and CVDs [5, 6]. However, there is controversy about which age intervals of accelerated growth (weight and height changes) lead to the development of chronic conditions. Evidence suggests that accelerated weight gain during infancy is associated with an adverse metabolic risk profile at adulthood [7-9]. In addition, it has been found that rapid weight gain increase the risk of metabolic disturbances when occurs during childhood, as a result of a more rapid development of body fat mass [10].

Studies on growth trajectories throughout life course and adult outcomes examine weight gain, without any distinction between the weight gain relative to height and linear growth. Dissimilar consequences of these measures have been found, which are relevant for healthcare policy makers when developing programs on cardiometabolic risk prevention. In this study, we aimed to assess the associations between size at birth (weight and length), conditional relative weight (CWh) and conditional length/height (CH) at ages 1, 4, 11, 15, and 18 years old and cardiometabolic risks markers (C-reactive protein (CRP), lipid profile, body mass index (BMI) and waist circumference (WC)) in adolescents aged 18 years old members of the 1993 Pelotas Birth Cohort study.

For peer review only - http://bmjopen.bmj.com/site/about/guidelines.xhtml

Methods

The city of Pelotas is located in Southern Brazil. It is a middle-sized city with nearly 330,000 inhabitants. In 1993, all mothers of hospital-delivered newborns (99% of all births) who resided in the urban area of the city were invited to participate in a birth cohort study. Data were collected on 5,249 live births and only 16 individuals refused to participate. The cohort participants have been followed up at different time points thereafter. All visits were carried out by trained interviewers and fieldwork team members. Further details of the methodology have been published previously [11].

This study included information from six follow-ups: at perinatal, and at ages 1-, 4-, 11-, 15- and 18-years. The 1- and 4-year old follow-ups were conducted only in a subsample of all low birthweight children plus a random sample of 20% of the rest of the sample (1460 children). The response rates were 99.6%, 93.4%, 87.2% 87.5%, 85.7% and 81.4%, respectively Household visits were performed in every follow-up except for the 18-y old wave that took place at the university research clinic, where interviews, physical exams and collection of biological samples were carried out [12].

The study and its protocols were approved by the School of Medicine Ethics Committee of the Federal University of Pelotas. All participants or their legal representatives voluntarily signed a consent letter (verbal consent was provided in perinatal phase) prior to participation in each follow-up.

Assessment of outcomes

For peer review only - http://bmjopen.bmj.com/site/about/guidelines.xhtml

We examined the following cardiometabolic risk markers measured at the 18-year old visit: CRP, total cholesterol (TC), HDL-cholesterol (HDL-C), LDL-cholesterol (LDL-C), triglycerides (TGL), systolic blood pressure (SBP), diastolic blood pressure (DBP), BMI and WC.

Random venous blood samples were collected, left at room temperature for 30 minutes and then centrifuged for 15 minutes at 2000g. Serum aliquots were stored at -80°C until analysis. The tests were not taken in pregnant or suspected pregnant. Lipids were measured using an automatic enzymatic colorimetric method in biochemistry analyzer BS-380, Mindray (Shenzhen Mindray Bio-Medical Electronics Co., Ltd, China). CPR was measured by turbidimetric immunoassay using also the BS-380 analyzer. Blood pressure was recorded in seated position using a calibrated digital wrist monitor (Omron HEM-629, Beijing, China). Measurements were taken at the start and at the end of the visit and mean of the two measurements was used in the analysis.

Current BMI was calculated by dividing weight in kilograms by height squared in meters. Weight was measured using a scale coupled to BodPod® equipment (Life Measurement, Inc., Concord, CA, USA) and height was obtained with standardized techniques using a wall-mounted stadiometer (SECA 240; Seca, Birmingham, United Kingdom). Waist circumference was measured with a fiberglass tape at the narrowest point of the torso.

Assessment of exposures

Birth weight and length were recorded in the maternity hospitals at delivery. In the subsequent follow-ups the measurements were conducted at participant's home and at the

For peer review only - http://bmjopen.bmj.com/site/about/guidelines.xhtml

research clinic in the 18-year old visit. On each occasion, weight and length or height were measured by trained field workers using standard equipment and protocols.

We studied growth patterns in several life periods: infancy (from birth to 1 year old), early and mid-childhood (1 to 4 and 4 to 11 years old, respectively), and early and late adolescence (11 to 15 and 15 to 18 years old, respectively). For each age interval, the separate effects of weight gain and linear growth were examined, using conditional relative weight and conditional length/height proposed by Adair et al [13]. Conditional relative weight takes into account current height and preceding weights and lengths or heights, and conditional length/height takes into account prior weight and length or height measures but not current weight [13].

To calculate these conditional measures, we computed sex-specific internal z-scores for weight and length or height at each follow-up. Then, we regressed the z-score size measurements (weight or height) at a given age, on z-scores at all previous measurements. The conditional measure is represented by the standardized residuals derived from the regression, and indicates the deviation from the individual's expected measures, in view of his or her previous growth and the average growth of the cohort members. This could be interpreted as a measure of relatively faster or slower weight or length/height change over a period of time. For example, an adolescence with a positive value in CWh from 11 to 15 years, put on greater weight compared with his or her previous weights and length/heights and weights and length/heights of all cohort participants. As the conditional variables are uncorrelated, they can be included in a multiple regression model without breaking any assumption of collinearity [14].

For peer review only - http://bmjopen.bmj.com/site/about/guidelines.xhtml

Statistical analyses

We first described the outcomes by means (SD) (geometric means for CRP and TGL). T-tests were used to estimate mean differences between sexes. To assess the association between each outcome and conditional growth we used linear regression and p-values were obtained by Wald's test. The outcomes were standardized to allow direct comparisons of the regression coefficients. We adjusted for following confounder factors: family income (in minimum wages), maternal education (completed years of schooling) at birth and self-reported skin color (white, black or others). Unadjusted and adjusted coefficients and statistical significance of associations did not differ markedly with the inclusion of confounders in our models, thus we presented only adjusted results. Analyses were performed using Stata 12.1 (Stata Corp., College Station, Texas, and EUA) and stratified by sex.

Results

At a mean age of 18.5 years 4106 adolescents were evaluated, of which 3869 had blood information, 3987 had blood pressure measured, 3977 had anthropometric exams. Conditional relative weight and conditional length/height data across infancy, childhood and adolescence were available for 957 participants. Our main analysis samples consisted of those cohort members who had complete data on exposures, confounders and at least one outcome. Therefore our samples size were 917 for blood exams, and 946 for blood pressure, BMI and WC.

Mean levels of SBP were higher in boys than girls, as well as WC. On the other hand, girls had higher values on TC, HDL-C, LDL-C and TGL compared to boys. No differences were shown for DBP and BMI by sex (Table 1).

For peer review only - http://bmjopen.bmj.com/site/about/guidelines.xhtml

Table 1. Mean (SD) for outcomes at 18 years of age in the main analyses samples, stratified by sex. 1993 Pelotas Birth Cohort[a]

**Outcomes**	Boys N=438	Girls N=479	P-value
Protein-c (mg/L) [b]	0.64 (3.10)	1.35 (3.92)	<0.001
Total cholesterol (mg/dl)	151.31 (24.46)	172.29 (30.35)	<0.001
HDL cholesterol (mg/dl)	52.78 (8.75)	59.50 (10.54)	<0.001
LDL cholesterol (mg/dl)	83.55 (18.46)	97.14 (25.42)	<0.001
Triglycerides (mg/dl) [b]	70.81 (1.46)	74.79 (1.44)	0.03
Systolic blood pressure (mm/Hg) [c]	130.35 (11.56)	115.40 (10.04)	<0.001
Diastolic blood pressure (mm/Hg) [c]	70.54 (8.29)	69.71 (7.83)	0.12
Body mass index [c]	23.00 (4.25)	23.58 (5.05)	0.05
Waist circumference [c]	77.51 (9.71)	73.74 (10.32)	<0.001

Data are arithmetic mean (SD) unless otherwise indicated

[a] Main analyses sample includes individuals with complete data on all growth measures, all confounders and at least one outcome.

[b] Geometric mean (SD)

[c] Boys N= 447 ; girls N=499

Mean outcome values of the main analysis samples were compared with mean outcome values of all participants who had information on blood tests, blood pressure and anthropometric measures. Only few differences were observed between both samples. Boys included in the main analyses had lower CPR and higher HDL-C values as compared to boys who were evaluated at the 18-years follow-up. Among females, those included in the main analyses showed higher LDL-C than the total girls who were examined at the 18-years follow-up (Supplementary file). Proportions of males and females were slightly different in both samples, with males slightly sub-represented in the main analyses sample compared with the sample comprised by all

For peer review only - http://bmjopen.bmj.com/site/about/guidelines.xhtml

participants who had information on blood tests, blood pressure and anthropometric measures (about 47.6% vs 49.8%).

Tables 2 and 3 show the association of size at birth, CWh and CH with cardiometabolic risk markers at 18 years old.

CRP levels showed positive association with CWh during childhood and adolescence (from 1 to 4 years, 4 to 11 years and 11 to 15 years in both sexes, and 15 to 18 years only in girls). The association between CRP and weight gain appeared to be stronger and with an increasing trend across age periods in girls. Positive associations between CRP and CH between 1 and 4 years were found in both sexes (Tables 2 and 3).

Overall, lipid profile was associated with CWh gain during childhood and adolescence, especially in boys. CWh in the early childhood (1 to 4 years) was positive associated only with TGL, and CWh between 4 and 11 years was positively associated with LDL-C and TGL and negatively associated with HDL-C (Table 2). CWh during early and late adolescence (11 to 15 years and 15 to 18 years) were positively related with TC, LDL-C and TGL, and negatively related with HDL-C(Table 2). In girls, HDL-C was inversely associated only with CWh in mid-childhood (4-11y) and both early and late adolescence (11-15y and 15-18y); whereas TC and LDL-C and TGL showed positive associations with CWh in late adolescence (Table 3).

Findings for SBP were similar for both sexes. However, DBP showed different patterns between sexes. Higher CWh during early and mid-childhood (1-4 y and 4-11y) and through early and late adolescence (11-15 y and 15-18y) was associated with higher SBP in both sexes, and with higher DBP only in girls. Regarding linear growth, conditional length gain during the first year of life and CH gain during early and mid-childhood (1-4 y and 4-11y) was related with

For peer review only - http://bmjopen.bmj.com/site/about/guidelines.xhtml

increased levels of SBP in both sexes, whereas CH gain during early and mid-childhood were related with increased levels of DBP only in girls (Tables 2 and 3).

CWh in all age periods were associated with higher BMI and WC in boys and girls, with apparently larger coefficients after the first year of life (Table 2 and 3). CH between birth and age 1 year were positive related with BMI and WC only in boys. CH throughout early and mid-childhood (1-4 y and 4-11y) was positive related with BMI and WC in both sexes (Table 2 and 3). CWh was more strongly related to BMI and WC than linear growth.

For peer review only

For peer review only - http://bmjopen.bmj.com/site/about/guidelines.xhtml

Table 2. Association of conditional relative weight and conditional height with cardiometabolic risk markers at 18 years old in boys. 1993 Pelotas Birth Cohort.

	Cardiometabolic risk markers								
	CRP	TC	HDL-C	LDL-C	TGL	SBP	DBP	BMI	WC
Conditional weight									
CWh 0-1 y	-0.00	0.07	0.03	0.07	0.01	0.04	0.04	0.24	0.26
	(-0.04; 0.04)	(-0.01; 0.14)	(-0.05; 0.10)	(-0.01; 0.14)	(-0.01; 0.03)	(-0.04; 0.11)	(-0.06; 0.13)	(0.17; 0.33)	(0.18; 0.35)
CWh 1-4 y	0.08	0.04	-0.03	0.05	0.04	0.09	0.06	0.43	0.41
	(0.03; 0.13)	(-0.04; 0.12)	(-0.11; 0.05)	(-0.03; 0.13)	(0.01; 0.06)	(0.01; 0.17)	(-0.04; 0.16)	(0.35; 0.51)	(0.33; 0.49)
CWh 4-11 y	0.09	0.06	-0.08	0.09	0.02	0.08	0.10	0.48	0.44
	(0.05; 0.14)	(-0.01; 0.13)	(-0.15; -0.01)	(0.01; 0.16)	(0.01; 0.04)	(0.01; 0.16)	(0.01; 0.19)	(0.42; 0.53)	(0.38; 0.50)
CWh 11-15 y	0.08	0.13	0.01	0.15	0.05	0.08	0.09	0.33	0.29
	(0.02; 0.13)	(0.05; 0.20)	(-0.07; 0.09)	(0.07; 0.23)	(0.02; 0.07)	(0.01; 0.15)	(-0.01; 0.19)	(0.28; 0.38)	(0.24; 0.36)
CWh 15-18 y	0.04	0.16	-0.11	0.18	0.07	0.12	0.08	0.46	0.47
	(-0.01; 0.09)	(0.08; 0.23)	(-0.19; -0.04)	(0.11; 0.26)	(0.05; 0.09)	(0.04; 0.19)	(-0.01; 0.18)	(0.44; 0.47)	(0.45; 0.50)
Conditional length/height									
CH 0-1 y	0.00	-0.01	-0.01	-0.00	-0.01	0.14	0.08	0.10	0.20
	(-0.04; 0.05)	(-0.10; 0.07)	(-0.09; 0.07)	(-0.09; 0.08)	(-0.03; 0.02)	(0.06; 0.23)	(-0.02; 0.18)	(0.01; 0.19)	(0.10; 0.29)
CH 1-4 y	0.05	0.06	0.03	0.05	0.02	0.09	0.04	0.18	0.24
	(0.01; 0.11)	(-0.02; 0.14)	(-0.05; 0.11)	(-0.04; 0.13)	(-0.01; 0.04)	(0.01; 0.18)	(-0.06; 0.14)	(0.09; 0.26)	(0.17; 0.33)
CH 4-11 y	-0.00	0.04	-0.08	0.07	0.02	0.13	0.21	0.13	0.18
	(-0.05; 0.04)	(-0.03; 0.12)	(-0.15; -0.01)	(-0.01; 0.15)	(-0.00; 0.04)	(0.05; 0.21)	(0.12; 0.30)	(0.07; 0.20)	(0.11; 0.25)
CH 11-15 y	-0.04	-0.00	0.01	0.00	-0.02	0.03	0.04	-0.01	0.02
	(-0.09; 0.01)	(-0.08; 0.07)	(-0.07; 0.09)	(-0.08; 0.08)	(-0.04; 0.00)	(-0.05; 0.11)	(-0.07; 0.14)	(-0.06; 0.04)	(-0.04; 0.08)
CH 15-18 y	-0.00	-0.10	-0.03	-0.08	-0.02	0.07	0.02	-0.05	0.02
	(-0.05; 0.05)	(-0.17; -0.02)	(-0.11; 0.04)	(-0.15; -0.00)	(-0.04; 0.00)	(-0.01; 0.15)	(-0.08; 0.12)	(-0.10; -0.01)	(-0.03; 0.08)

For peer review only

CRP: reactive-C protein, TC: total cholesterol, HDL-C: high-density lipoprotein cholesterol, LDL-C: low-density lipoprotein cholesterol, TGL: triglycerides, DBP: diastolic blood pressure, SBP: systolic blood pressure, BMI: body mass index, CC: waist circumference, CWh: conditional relative weight, CH: conditional height

Data are β (95% CI). The outcome variables were normalized. Regression coefficient (β) values were calculated with linear regression models and indicate the SD change in the outcome per SD change in the predictor. All models were adjusted for mother's education (years of schooling) and household wealth (in minimum wages) at birth and skin color of the adolescent.

For peer review only - http://bmjopen.bmj.com/site/about/guidelines.xhtml

Table 3. Association of conditional relative weight and conditional height with cardiometabolic risk markers at 18 years old in girls. 1993 Pelotas Birth Cohort

	Cardiometabolic irsk markers								
	CRP	TC	HDL-C	LDL-C	TGL	SBP	DBP	BMI	WC
Conditional weight									
CWh 0-1 y	0.03	-0.01	-0.04	-0.01	0.01	0.06	0.03	0.25	0.21
	(-0.05; 0.12)	(-0.10; 0.09)	(-0.13; 0.05)	(-0.11; 0.10)	(-0.03; 0.06)	(-0.00; 0.13)	(-0.06; 0.12)	(0.15; 0.35)	(0.12; 0.30)
CWh 1-4 y	0.08	-0.02	-0.02	-0.03	0.03	0.12	0.12	0.56	0.45
	(-0.01; 0.18)	(-0.12; 0.08)	(-0.11; 0.08)	(-0.14; 0.07)	(-0.03; 0.08)	(0.05; 0.18)	(0.03; 0.23)	(0.47; 0.65)	(0.37; 0.54)
CWh 4-11 y	0.12	-0.04	-0.15	0.03	0.00	0.10	0.13	0.62	0.53
	(0.03; 0.20)	(-0.13; 0.06)	(-0.24; -0.07)	(-0.07; 0.13)	(-0.02; 0.02)	(0.04; 0.17)	(0.04; 0.22)	(0.55; 0.68)	(0.47; 0.59)
CWh 11-15 y	0.14	0.00	-0.18	0.05	0.03	0.15	0.11	0.45	0.39
	(0.06; 0.24)	(-0.09; 0.01)	(-0.27; -0.10)	(-0.05; 0.15)	(0.01; 0.05)	(0.09; 0.21)	(0.03; 0.20)	(0.40; 0.50)	(0.34; 0.46)
CWh 15-18 y	0.21	0.14	-0.10	0.20	0.03	0.16	0.13	0.51	0.47
	(0.12; 0.30)	(0.04; 0.24)	(-0.19; -0.02)	(0.09; 0.29)	(0.01; 0.05)	(0.10; 0.22)	(0.04; 0.22)	(0.50; 0.53)	(0.43; 0.50)
Conditional length/height									
CH 0-1 y	0.02	0.08	0.17	0.03	-0.02	0.09	0.01	0.01	0.08
	(-0.07; 0.13)	(-0.01; 0.17)	(0.08; 0.26)	(-0.07; 0.12)	(-0.07; 0.02)	(0.02; 0.15)	(-0.07; 0.10)	(-0.08; 0.11)	(-0.00; 0.17)
CH 1-4 y	0.13	0.06	0.11	0.01	0.03	0.10	0.11	0.12	0.22
	(0.03; 0.22)	(-0.04; 0.16)	(0.02; 0.21)	(-0.09; 0.12)	(-0.02; 0.08)	(0.03; 0.17)	(0.01; 0.20)	(0.01; 0.22)	(0.12; 0.31)
CH 4-11 y	-0.02	0.05	0.04	0.04	-0.00	0.08	0.20	0.12	0.12
	(-0.11; 0.07)	(-0.04; 0.15)	(-0.04; 0.13)	(-0.06; 0.14)	(-0.02; 0.02)	(0.02; 0.14)	(0.11; 0.29)	(0.04; 0.20)	(0.04; 0.20)
CH 11-15 y	-0.01	-0.02	-0.07	0.02	-0.01	0.08	0.02	-0.01	0.08
	(-0.10; 0.08)	(-0.11; 0.08)	(-0.15; 0.02)	(-0.09; 0.12)	(-0.03; 0.02)	(0.02; 0.15)	(-0.07; 0.10)	(-0.07; 0.06)	(0.01; 0.13)
CH 15-18 y	-0.10	0.00	-0.05	0.01	0.01	-0.00	0.00	-0.05	-0.01
	(-0.18; -0.01)	(-0.09; 0.10)	(-0.14; 0.03)	(-0.09; 0.11)	(-0.01; 0.03)	(-0.07; 0.06)	(-0.08; 0.09)	(-0.07; -0.03)	(-0.05; 0.05)

CRP: reactive-C protein, TC: total cholesterol, HDL-C: high-density lipoprotein cholesterol, LDL-C: low-density lipoprotein cholesterol, TGL: triglycerides, DBP: diastolic blood pressure, SBP: systolic blood pressure, BMI: body mass index, CC: waist circumference, CWh: conditional relative weight , CH : conditional height

Data are β (95% CI). The outcome variables were normalized. Regression coefficient (β) values were calculated with linear regression models and indicate the SD change in the outcome per SD change in the predictor. All models were adjusted for mother's education (years of schooling), household wealth (in minimum wages) at birth and skin color of the adolescent.

For peer review only - http://bmjopen.bmj.com/site/about/guidelines.xhtml

Discussion

Our results showed some differences in the relative contributions of weight gain and linear growth to cardiometabolic risk. We observed that higher conditional relative weight at most age periods in childhood (1-4 y and 4-11 y) and adolescence (11-15 y and 15-18 y) was positively associated with most of the cardiometabolic markers (CRP, lipid profile, SBP, DBP, BMI and WC) at 18 years old adolescents of both sexes, while conditional relative weight during the first year of life was only related to BMI and WC. By contrast, associations between linear growth and the cardiometabolic markes showed a less consistent pattern when compared with weight gain. Greater conditional length at infancy was associated with higher values of SBP in boys and girls, and with BMI and WC only in boys.

Overall, our findings are consistent with published literature of low-, middle- and high-income countries showing that excessive weight gain from mid-childhood onwards, specifically after the second year of life, predicts metabolic and cardiovascular diseases later in life [13, 15, 16]. The association between weight gain throughout life and CRP concentrations at young adulthood was examined in the 1982 Pelotas Birth Cohort. In agreement with our analysis, the study showed that excessive weight gain at all ages periods after the second year of life in both sexes were positively associated with CRP levels in 23 years old participants of both sexes [17]. We also found that excessive weight gain during childhood and adolescence was associated with increased total cholesterol, LDL-C and TGL and decreased HDL-C. Weight gain from birth to 4 years and lipid profile levels at 18 years was studied in the 1982 Cohort and negative associations were found between excessive weight gain from 2 to 4 years and HDL-C, although the association was reduced after controlling to current BMI [18]. In a study using data from the

For peer review only - http://bmjopen.bmj.com/site/about/guidelines.xhtml

ALSPAC cohort in England, the authors observed that greater BMI in mid-childhood predicted higher blood pressure (SBP and DBP) in 17 years old adolescents [19].

Fall et al., assessed the relations between components of metabolic syndrome and weight gain at three periods from birth to age 28 years, age in which the outcomes were measured. In line with our findings, the study showed that greater weight gain in childhood (2 to 11 years) was associated with increased values of WC, SBP and TLG, while greater weight gain from 11 to 28 years predicted higher WC, TGL, total cholesterol and SBP and lower HDL-C. They also observed positive associations between rapid weight gain during the two first postnatal years and WC, SBP and TGL [15].

In concordance with analysis from the Vellore Birth Cohort in India [20], we observed that infancy (birth to 1 year) rapid conditional relative weight was positive associated with BMI and WC, but not with other cardiometabolic markers. A meta-analyses assessing infant growth and subsequent obesity, also showed that infants who grew more rapidly had higher risk of developing obesity at posterior ages [7]. Studies that assessed the relation between growth and body composition showed that rapid weight gain in infancy were more related with fat free mass than fat mass in adulthood [13, 21]. In contrast, rapid weight gain from childhood onwards generally is associated with accumulation of greater fat mass compared with free fat mass [10, 13, 21-23]. These findings may explain the positive associations of relative weight gain at all age periods with BMI, as this indicator does not distinguish body fat from free fat mass.

In relation to linear growth, we observed that faster conditional height mainly in childhood was found to be positive associated with blood pressure (SBP and DBP), BMI and WC at 18 years old in both sexes, with less consistent associations in girls compared to boys. A previous report with data of the same cohort examined the association between conditional

For peer review only - http://bmjopen.bmj.com/site/about/guidelines.xhtml

growth at three different age ranges up to 4 years old, BMI and blood pressure at 15 years, and found positive associations between conditional height from 1 to 4 years and both outcomes, although the association with SBP became insignificant adjusted for current BMI [24]. Haugaard et al, found positive association between linear growth during childhood and SBP and WC at age 8 years old [25]. The previously mentioned Vellore Birth Cohort showed that rapid conditional height throughout course life was positive associated with blood pressure and WC in young adults [20]. It is known that blood pressure is higher in taller people, this may be result of an adaptation of the vascular function to perfuse a longer arterial tree. However, inverse relations between adult height and cardiovascular diseases have been described as well [26], which suggests that this adaption possibly has not pathological consequences.

Strengths of this study include the large and population based sample and the availability of several anthropometric and biological markers of cardiometabolic risk. Furthermore, our data have been collected prospectively since birth by trained staff with the use of standardized methods, reducing the susceptibility to misclassification. We also highlight the use of conditional growth to examine highly correlated measurements typical of longitudinal studies, and the assessment of the separate contributions of linear growth and weight gain relative to linear growth. Nevertheless, the assessment of relative weight gain does not distinguish between free-fat and fat mass gain.

We acknowledge some limitations of our study. First, at 18 years old, we followed up 81.3% of the original cohort and managed to have outcome measurements among most of them (94.2%, 97.1% and 97.8% for blood exams, anthropometric measures and blood pressure, respectively). These are high follow-ups rates for longitudinal studies and minimize the possibility of selection bias, however, our analysis were carried out only by those cohort

For peer review only

members with anthropometric data from several follow-ups (including sub-samples). The potential impact of the losses on our results is difficult to assess. Second, we cannot rule out the possibility of random significant associations, as it is known that multiple hypothesis testing lead to increased Type I error. Third, measurements at age 2 years old were not available in the 1993 Pelotas Birth Cohort, and therefore we had no ability to assess growth at this age point. The inclusion of age 2 years old in longitudinal studies assessing the associations between growth and cardiometabolic risk is very important due to growing evidence showing that excessive relative weight gain denotes increased risk for cardiometabolic health when occurred after the age of 2 years. For this reason, our findings support the initiative of improving nutrition during the "first 1000 days of life" (from conception up to age 2 years) to promote long-term benefits on health, as greater infant weight gain and linear growth have more benefits than risks for health, specially improvement of capital human outcomes as schooling and final achieved height [13, 16].

Given that cardiometabolic risk can track from adolescence to adulthood, a better understanding of the possible adverse effects of growth patterns at earlier stages in life can help to develop interventions aimed at preventing subsequent chronic diseases. Based on this study and other published evidence, we conclude that excessive weight gain from childhood onward may have an adverse effect on cardiometabolic health later in life. This reinforces efforts to inform strategies to avoid putting on weight children after their 2 first years of life for cardiovascular prevention. Evidence regarding linear growth throughout birth to late adolescence and subsequent cardiometabolic risk remains unclear and needs further investigation.

Contributorship: RB, MCFA and MCR-M designed the study. RB performed the analysis. RB, MCR-M, MCFA and VMS contributed to the interpretation of the results and critical revision of

For peer review only - http://bmjopen.bmj.com/site/about/guidelines.xhtml

the manuscript. HDG, AMM, IOO, MCFA participated in the design and conduct of the original cohort studies as well as in interpreting results and reviewing the manuscript. RB wrote the manuscript, and all authors contributed to and approved the final version.

Competing interests: None declared

Funding: This article is based on data from the study "Pelotas Birth Cohort, 1993" conducted by Postgraduate Program in Epidemiology at Universidade Federal de Pelotas with the collaboration of the Brazilian Public Health Association (ABRASCO). From 2004 to 2013, the Wellcome Trust supported the 1993 birth cohort study. The European Union, National Support Program for Centers of Excellence (PRONEX), the Brazilian National Research Council (CNPq), and the Brazilian Ministry of Health supported previous phases of the study.

Ethical approval: The study and its protocols were approved by the School of Medicine Ethics Committee of the Federal University of Pelotas. All participants or their legal representatives voluntarily signed a consent letter (verbal consent was provided in perinatal phase) prior to participation in each follow-up.

Acknowledgements: We are grateful to all the adolescents who took part in the Pelotas birth cohorts, and the Pelotas teams, including research scientists, interviewers, workers and volunteers.

Data sharing: Due to confidentiality restrictions related to the ethics approval for this study, no identifying information about participants may be released. As recipients, the authors were allowed to publish analytic results from the data, but not the data itself, due to confidentiality conditions.

For peer review only - http://bmjopen.bmj.com/site/about/guidelines.xhtml

References

1 WHO. Global Health Risks: mortality and burden of disease atrributable to selected major risks. 2009.

2 Wells JC. The programming effects of early growth. *Early human development* 2007;**83**:743-8.

3 Whincup PH, Kaye SJ, Owen CG*, et al.* Birth weight and risk of type 2 diabetes: a systematic review. *Jama* 2008;**300**:2886-97.

4 Newsome CA, Shiell AW, Fall CH*, et al.* Is birth weight related to later glucose and insulin metabolism?--A systematic review. *Diabetic medicine : a journal of the British Diabetic Association* 2003;**20**:339-48.

5 Eriksson JG. Early growth and coronary heart disease and type 2 diabetes: findings from the Helsinki Birth Cohort Study (HBCS). *The American journal of clinical nutrition* 2011;**94**:1799S-802S.

6 Barker DJ. Fetal programming of coronary heart disease. *Trends in endocrinology and metabolism: TEM* 2002;**13**:364-8.

7 Baird J, Fisher D, Lucas P*, et al.* Being big or growing fast: systematic review of size and growth in infancy and later obesity. *BMJ (Clinical research ed)* 2005;**331**:929.

8 Monteiro PO, Victora CG. Rapid growth in infancy and childhood and obesity in later life--a systematic review. *Obesity reviews : an official journal of the International Association for the Study of Obesity* 2005;**6**:143-54.

9 Ong KK, Ahmed ML, Emmett PM*, et al.* Association between postnatal catch-up growth and obesity in childhood: prospective cohort study. *BMJ (Clinical research ed)* 2000;**320**:967-71.

10 Reilly JJ, Armstrong J, Dorosty AR*, et al.* Early life risk factors for obesity in childhood: cohort study. *BMJ (Clinical research ed)* 2005;**330**:1357.

11 Victora CG, Hallal PC, Araujo CL*, et al.* Cohort profile: the 1993 Pelotas (Brazil) birth cohort study. *International journal of epidemiology* 2008;**37**:704-9.

12 Goncalves H, Assuncao MC, Wehrmeister FC*, et al.* Cohort profile update: The 1993 Pelotas (Brazil) birth cohort follow-up visits in adolescence. *International journal of epidemiology* 2014;**43**:1082-8.

13 Adair LS, Fall CH, Osmond C*, et al.* Associations of linear growth and relative weight gain during early life with adult health and human capital in countries of low and middle income: findings from five birth cohort studies. *Lancet* 2013;**382**:525-34.

14 Keijzer-Veen MG, Euser AM, van Montfoort N*, et al.* A regression model with unexplained residuals was preferred in the analysis of the fetal origins of adult diseases hypothesis. *J Clin Epidemiol* 2005;**58**:1320-4.

15 Fall CH, Sachdev HS, Osmond C*, et al.* Adult metabolic syndrome and impaired glucose tolerance are associated with different patterns of BMI gain during infancy: Data from the New Delhi Birth Cohort. *Diabetes Care* 2008;**31**:2349-56.

16 Victora CG, Adair L, Fall C*, et al.* Maternal and child undernutrition: consequences for adult health and human capital. *Lancet* 2008;**371**:340-57.

17 Nazmi A, Gonzalez DC, Oliveira IO*, et al.* Life course weight gain and C-reactive protein levels in young adults: findings from a Brazilian birth cohort. *American journal of human biology : the official journal of the Human Biology Council* 2009;**21**:192-9.

For peer review only - http://bmjopen.bmj.com/site/about/guidelines.xhtml

18 Horta BL, Victora CG, Lima RC*, et al.* Weight gain in childhood and blood lipids in adolescence. *Acta paediatrica (Oslo, Norway : 1992)* 2009;**98**:1024-8.
19 Howe LD, Chaturvedi N, Lawlor DA*, et al.* Rapid increases in infant adiposity and overweight/obesity in childhood are associated with higher central and brachial blood pressure in early adulthood. *J Hypertens* 2014;**32**:1789-96.
20 Antonisamy B, Vasan SK, Geethanjali FS*, et al.* Weight Gain and Height Growth during Infancy, Childhood, and Adolescence as Predictors of Adult Cardiovascular Risk. *J Pediatr* 2016.
21 Victora CG, Sibbritt D, Horta BL*, et al.* Weight gain in childhood and body composition at 18 years of age in Brazilian males. *Acta paediatrica (Oslo, Norway : 1992)* 2007;**96**:296-300.
22 Wells JC, Hallal PC, Wright A*, et al.* Fetal, infant and childhood growth: relationships with body composition in Brazilian boys aged 9 years. *International journal of obesity (2005)* 2005;**29**:1192-8.
23 Sachdev HS, Fall CH, Osmond C*, et al.* Anthropometric indicators of body composition in young adults: relation to size at birth and serial measurements of body mass index in childhood in the New Delhi birth cohort. *The American journal of clinical nutrition* 2005;**82**:456-66.
24 Menezes AM, Hallal PC, Dumith SC*, et al.* Adolescent blood pressure, body mass index and skin folds: sorting out the effects of early weight and length gains. *Journal of epidemiology and community health* 2012;**66**:149-54.
25 Haugaard LK, Baker JL, Perng W*, et al.* Growth in Total Height and Its Components and Cardiometabolic Health in Childhood. *Plos One* 2016;**11**:e0163564.
26 Wannamethee SG, Shaper AG, Whincup PH*, et al.* Adult height, stroke, and coronary heart disease. *Am J Epidemiol* 1998;**148**:1069-76.

For peer review only

For peer review only - http://bmjopen.bmj.com/site/about/guidelines.xhtml

Supplementary file

Table 1. Mean (SD) for outcomes at 18 years of age in the main analyses samples* and samples comprised by all the adolescents with outcomes measures at the 18 years follow-up, stratified by sex. 1993 Pelotas Birth Cohort.

Outcomes	Boys					Girls				
	N	Main analyses sample	N	All participants	p-value*	N	Main analyses sample	N	All participants	p-value*
Plasma glucose (mg/dl)	438	93.60 (20.88)	1933	93.92 (22.65)	0.79	479	88.41 (15.36)	1936	89.74 (18.64)	0.15
HbA1c (%)	438	4.97 (0.60)	1924	4.96 (0.57)	0.74	475	4.86 (0.50)	1910	4.84 (0.52)	0.45
C-reactive Protein (mg/L)	438	0.64 (3.10)	1933	0.67 (3.26)	<0.01	479	1.35 (3.92)	1936	1.35 (3.86)	0.88
Total cholesterol (mg/dl)	438	151.31 (24.46)	1933	152.72 (24.55)	0.27	479	172.29 (30.35)	1936	169.80 (29.19)	0.10
HDL cholesterol (mg/dl)	438	52.78 (8.75)	1933	51.78 (8.75)	0.03	479	59.50 (10.54)	1936	59.84 (10.92)	0.54
LDL cholesterol (mg/dl)	438	83.55 (18.46)	1933	84.28 (20.23)	0.49	479	97.14 (25.42)	1936	93.89 (23.84)	0.01
Triglycerides (mg/dl)	438	70.81 (1.46)	1933	73.56 (1.51)	0.16	479	74.79 (1.44)	1936	74.93 (1.49)	0.62
Systolic blood pressure (mm/Hg)	447	130.3 (11.56)	1979	130.71 (11.90)	0.52	499	115.40 (10.04)	2008	115.06 (9.95)	0.50
Diastolic blood pressure (mm/Hg)	447	70.54 (8.29)	1979	70.95 (7.94)	0.33	499	69.64 (7.83)	2008	69.46 (7.75)	0.64
Body mass index	447	22.99 (4.24)	1970	23.36 (4.23)	0.10	499	23.58 (5.06)	2003	23.52 (4.76)	0.80
Waist circumference (cm)	447	77.51 (9.71)	1972	78.45 (9.61)	0.06	499	73.74 (10.31)	2005	73.75 (9.75)	0.98

Data are arithmetic mean (SD) unless otherwise indicated
* Geometric mean (SD)
*Main analyses samples includes individuals with complete data on all growth measures, all confounders and at least one outcome.
*p-value for T-test. C-reactive protein and triglycerides were log transformed to performed de test.

For peer review only

For peer review only

STROBE Statement—checklist of items that should be included in reports of observational studies

	Item No	Recommendation	Pag.
**Title and abstract**	1	(*a*) Indicate the study's design with a commonly used term in the title or the abstract	1
		(*b*) Provide in the abstract an informative and balanced summary of what was done and what was found	1
**Introduction**			
Background/rationale	2	Explain the scientific background and rationale for the investigation being reported	4
Objectives	3	State specific objectives, including any prespecified hypotheses	4
**Methods**			
Study design	4	Present key elements of study design early in the paper	5
Setting	5	Describe the setting, locations, and relevant dates, including periods of recruitment, exposure, follow-up, and data collection	5
Participants	6	(*a*) *Cohort study*—Give the eligibility criteria, and the sources and methods of selection of participants. Describe methods of follow-up<br>*Case-control study*—Give the eligibility criteria, and the sources and methods of case ascertainment and control selection. Give the rationale for the choice of cases and controls<br>*Cross-sectional study*—Give the eligibility criteria, and the sources and methods of selection of participants	5
		(*b*) *Cohort study*—For matched studies, give matching criteria and number of exposed and unexposed<br>*Case-control study*—For matched studies, give matching criteria and the number of controls per case	NA
Variables	7	Clearly define all outcomes, exposures, predictors, potential confounders, and effect modifiers. Give diagnostic criteria, if applicable	6-7
Data sources/ measurement	8*	For each variable of interest, give sources of data and details of methods of assessment (measurement). Describe comparability of assessment methods if there is more than one group	6-7
Bias	9	Describe any efforts to address potential sources of bias	NA
Study size	10	Explain how the study size was arrived at	8
Quantitative variables	11	Explain how quantitative variables were handled in the analyses. If applicable, describe which groupings were chosen and why	8
Statistical methods	12	(*a*) Describe all statistical methods, including those used to control for confounding	8
		(*b*) Describe any methods used to examine subgroups and interactions	NA
		(*c*) Explain how missing data were addressed	8
		(*d*) *Cohort study*—If applicable, explain how loss to follow-up was addressed<br>*Case-control study*—If applicable, explain how matching of cases and controls was addressed<br>*Cross-sectional study*—If applicable, describe analytical methods taking account of sampling strategy	8
		(*e*) Describe any sensitivity analyses	

For peer review only

Continued on next page

For peer review only

For peer review only - http://bmjopen.bmj.com/site/about/guidelines.xhtml

**Results**			
Participants	13*	(a) Report numbers of individuals at each stage of study—eg numbers potentially eligible, examined for eligibility, confirmed eligible, included in the study, completing follow-up, and analysed	8
		(b) Give reasons for non-participation at each stage	NA
		(c) Consider use of a flow diagram	NA
Descriptive data	14*	(a) Give characteristics of study participants (eg demographic, clinical, social) and information on exposures and potential confounders	9
		(b) Indicate number of participants with missing data for each variable of interest	8
		(c) *Cohort study*—Summarise follow-up time (eg, average and total amount)	
Outcome data	15*	*Cohort study*—Report numbers of outcome events or summary measures over time	*Table 1*
		Case-control study—Report numbers in each exposure category, or summary measures of exposure	
		Cross-sectional study—Report numbers of outcome events or summary measures	
Main results	16	(*a*) Give unadjusted estimates and, if applicable, confounder-adjusted estimates and their precision (eg, 95% confidence interval). Make clear which confounders were adjusted for and why they were included	Table 1 and 3
		(*b*) Report category boundaries when continuous variables were categorized	
		(*c*) If relevant, consider translating estimates of relative risk into absolute risk for a meaningful time period	
Other analyses	17	Report other analyses done—eg analyses of subgroups and interactions, and sensitivity analyses	
**Discussion**			
Key results	18	Summarise key results with reference to study objectives	14
Limitations	19	Discuss limitations of the study, taking into account sources of potential bias or imprecision. Discuss both direction and magnitude of any potential bias	16
Interpretation	20	Give a cautious overall interpretation of results considering objectives, limitations, multiplicity of analyses, results from similar studies, and other relevant evidence	14-17
Generalisability	21	Discuss the generalisability (external validity) of the study results	16
**Other information**			
Funding	22	Give the source of funding and the role of the funders for the present study and, if applicable, for the original study on which the present article is based	18

*Give information separately for cases and controls in case-control studies and, if applicable, for exposed and unexposed groups in cohort and cross-sectional studies.

Note: An Explanation and Elaboration article discusses each checklist item and gives methodological background and published examples of transparent reporting. The STROBE checklist is best used in conjunction with this article (freely available on the Web sites of PLoS Medicine at http://www.plosmedicine.org/, Annals of Internal Medicine at http://www.annals.org/, and Epidemiology at http://www.epidem.com/). Information on the STROBE Initiative is available at www.strobe-statement.org.

For peer review only

BMJ Open

Growth across life course and cardiovascular risk markers in 18 years old adolescents: the 1993 Pelotas Birth Cohort

Journal:	*BMJ Open*
Manuscript ID	bmjopen-2017-019164.R1
Article Type:	Research
Date Submitted by the Author:	25-Oct-2017
Complete List of Authors:	Buffarini, Romina; Universidade Federal de Pelotas<br>Restrepo-Méndez, Maria Clara; Universidade Federal de Pelotas, Centro Internacional de Equidade<br>Silveira, Vera; Federal University of Pelotas (UFPel), Postgraduate Programme in Epidemiology<br>Goncalves, Helen; Universidade Federal de Pelotas<br>Oliveira, Isabel; Federal University of Pelotas (UFPel), Postgraduate Programme in Epidemiology<br>Menezes, Ana; Federal University of Pelotas (UFPel), Postgraduate Programme in Epidemiology<br>Assuncao, M.C; Federal University of Pelotas, Post-Graduate Program in Epidemiology
**Primary Subject Heading**:	Epidemiology
Secondary Subject Heading:	Public health, Epidemiology
Keywords:	DIABETES & ENDOCRINOLOGY, EPIDEMIOLOGY, PAEDIATRICS, PUBLIC HEALTH

SCHOLARONE™
Manuscripts

Growth across life course and cardiovascular risk markers in 18 years old adolescents: the 1993 Pelotas Birth Cohort

Romina Buffarini[*1]; María Clara Restrepo-Méndez[2]; Vera Maria Silveira[3]; Helen D. Gonçalves[1], Isabel O. Oliveira[4], Ana Maria Menezes[1], Maria Cecília Formoso Assunção[5]

[1] Post-graduate Program in Epidemiology, Federal University of Pelotas, Marechal Deodoro 1160, 3rd floor, 96020-220, Pelotas, Brazil

[2] MRC Integrative Epidemiology Unit, Population Health Sciences, Bristol Medical School, University of Bristol, Oakfield House, Oakfield Grove, Bristol BS82BN, UK

[3] Clinical Medical Department, Faculty of Medicine, Federal University of Pelotas, Rua Félix da Cunha 614, Centro, 96010-000, Pelotas, Brazil

[4] Department of Physiology and Pharmacology, Federal University of Pelotas, Campus Universitário s/n, Capão do Leão, 96010-900, Pelotas, Brazil

[5] Department of Nutrition, School of Nutrition, Federal University of Pelotas, Campus Anglo, Gomes Carneiro 1, 96010-900, Pelotas , Brazil

[*]**Corresponding author:** Romina Buffarini. Correspondence address: Marechal Deodoro 1160, 3rd floor, 96020-220, Pelotas, RS, Brasil. Phone/Fax +55 (53) 3284 – 1300. E-mail: romibuffarini@gmail.com.

Manuscript word count: 3148

Abstract word count: 257

For peer review only

For peer review only - http://bmjopen.bmj.com/site/about/guidelines.xhtml

Abstract

Objective: We aimed to evaluate the association between growth trajectories from birth to adolescence and cardiovascular risk markers levels at age 18 years in a population based cohort. In order to disentangle the effect of weight gain from that of height gain, growth was analyzed using conditional weight relative to linear growth (CWh) and conditional length/height (CH).

Design: Prospective study.

Setting: 1993 Pelotas Birth Cohort, Southern Brazil.

Participants: Individuals that have been followed-up from birth up to adolescence (at birth, 1, 4, 11, 15 and 18 years).

Primary outcome measures: C-reactive protein (CRP), total cholesterol (TC), LDL-C, HDL-C, triglycerides (TGL), systolic and diastolic blood pressure (SBP and DBP), BMI and waist circumference (WC).

Results: In both sexes, greater CWh at 1 year is positively associated with BMI and WC, whereas greater CWh at most age periods in childhood and adolescence predicted higher CRP, TC, LDL-C, TGL, SBP, DBP, BMI and WC levels, as well as lower HDL-C level. Higher CH during infancy and childhood is positively related with SBP in boys and girls, and with BMI and WC only in boys.

Conclusion: Our study shows that rapid weight gain from 1 year old onwards is positively associated with several markers of cardiovascular risk at 18 years. Overall, our results for the first year of life add evidence to the "first 1000 days initiative" suggesting that prevention of excessive weight gain in childhood might be important in reducing subsequent cardiovascular risk.

Key-words: conditional growth, relative weight gain, linear growth, adolescents, cardiovascular risk, cohort studies.

For peer review only

Strengths and limitations:

- Population based sample birth cohort. Data collected prospectively over 18 years since birth with high follow ups rates.
- Availability of several anthropometric and biological markers of cardiovascular risk
- Use of conditional growth to examine highly correlated measurements, and assessment of the separate contributions of linear growth and relative weight gain.
- Use of sub-sample in one of the follow-ups. Multiple hypothesis testing lead to increased Type I error.
- Lack of anthropometric data at two years is a limitation in our study, since there is evidence suggesting that the hazards of rapid weight gain appear particularly after the two first years of life.

Funding: This article is based on data from the study "Pelotas Birth Cohort, 1993" conducted by Postgraduate Program in Epidemiology at Universidade Federal de Pelotas with the collaboration of the Brazilian Public Health Association (ABRASCO). From 2004 to 2013, the Wellcome Trust supported the 1993 birth cohort study. The European Union, National Support Program for Centers of Excellence (PRONEX), the Brazilian National Research Council (CNPq), and the Brazilian Ministry of Health supported previous phases of the study.

Competing interests: None declared

For peer review only

For peer review only - http://bmjopen.bmj.com/site/about/guidelines.xhtml

Introduction

Metabolic and cardiovascular diseases (CVDs) are important public health problems that cause enormous costs in terms of human and economic resources and are responsible for high rates of morbidity and mortality in most regions of the world[1].

Growth trajectories throughout life course, including the fetal period, may have effects on cardiovascular risk profile later in life [2]. It is well established in the literature that low birthweight (a marker of fetal growth restriction) is a risk factor for type 2 diabetes [3], insulin resistance [4] and CVDs [5, 6]. However, the critical age interval (first year of life or middle childhood) for accelerated growth (weight and height changes) that leads to the development of chronic conditions remains controversial [5, 7, 8]. Evidence suggests that accelerated weight gain during infancy is associated with an adverse metabolic risk profile at adulthood [7, 9, 10]. In addition, it has been found that rapid weight gain increase the risk of metabolic disturbances when occurs during childhood, as a result of a more rapid development of body fat mass [11]. The definition of this critical period is crucial to inform health policies.

Studies on growth trajectories throughout life course and adult outcomes examine weight gain, without any distinction between the weight gain relative to height and linear growth. Dissimilar consequences of these measures have been found [12, 13], which are relevant for healthcare policy makers when developing programs on cardiometabolic risk prevention. In this study, we aimed to assess the associations between size at birth (weight and length), conditional relative weight (CWh) and conditional length/height (CH) at ages 1, 4, 11, 15, and 18 years old and cardiovascular risks markers (C-reactive protein (CRP), lipid profile, body mass index

For peer review only

For peer review only - http://bmjopen.bmj.com/site/about/guidelines.xhtml

(BMI) and waist circumference (WC) in adolescents aged 18 years old members of the 1993 Pelotas Birth Cohort study.

Methods

The city of Pelotas is located in Southern Brazil. It is a middle-sized city with nearly 330,000 inhabitants. In 1993, all mothers who delivered a newborn in any of the five hospitals of the city and who resided in the urban area were invited to participate in a birth cohort study (99% of all births). Data were collected on 5,249 live births and only 16 individuals refused to participate. The cohort participants have been followed up at different time points thereafter. All visits were carried out by trained interviewers and fieldwork team members. Further details of the methodology have been published previously [14].

This study included information from six follow-ups: at perinatal, and at ages 1-, 4-, 11-, 15- and 18-years. The 1- and 4-year old follow-ups were conducted only in a subsample of the original cohort. Same children were the target population (N=1460) for both follow-ups which consisted of all low birthweight children (N= 510) plus a random sample of 20% of children who were not born low birth weight (N= 950). The response rates for each follow-up were 99.6% (N=5,249), 93.4% (N=1,363), 87.2% (N=1,273), 87.5% (N=4,452), 85.7% (N=4,349) and 81.4% (N=4,106), respectively (Supplementary file 1). More details of each follow up are described elsewhere [15]. A diagram with the description of the cohort follow-ups used in this study is available in Supplementary file1. Household visits were performed in every wave except for the 18-y old visit that took place at the university research clinic, where interviews, physical exams and collection of biological samples were carried out [16].

For peer review only - http://bmjopen.bmj.com/site/about/guidelines.xhtml

The protocol study was approved by the School of Medicine Ethics Committee of the Federal University of Pelotas. All participants or their legal representatives voluntarily signed a consent letter prior to participation in each follow-up. Verbal consent was provided in perinatal phase.

For peer review only

Assessment of outcomes

We examined the following cardiovascular risk markers measured at the 18-year old visit: CRP, total cholesterol (TC), HDL-cholesterol (HDL-C), LDL-cholesterol (LDL-C), triglycerides (TGL), systolic blood pressure (SBP), diastolic blood pressure (DBP), BMI and WC.

Venous blood samples were collected regardless of fasting status, left at room temperature for 30 minutes and then centrifuged for 15 minutes at 2000g. Serum aliquots were stored at -80°C until analysis. Blood samples were not taken in pregnant or suspected pregnant participants (N=59). Lipids were measured using an automatic enzymatic colorimetric method in biochemistry analyzer BS-380, Mindray (Shenzhen Mindray Bio-Medical Electronics Co., Ltd, China). CPR was measured by turbidimetric immunoassay using also the BS-380 analyzer. Blood pressure was recorded in seated position using a calibrated digital wrist monitor (Omron HEM-629, Beijing, China). Measurements were taken at the start and at the end of the visit and mean of the two measurements was used in the analysis.

Current BMI was calculated by dividing weight in kilograms by height squared in meters. Weight was measured using a scale coupled to BodPod® equipment (Life Measurement, Inc.,

For peer review only - http://bmjopen.bmj.com/site/about/guidelines.xhtml

Concord, CA, USA) and height was obtained with standardized techniques using a wall-mounted stadiometer (SECA 240; Seca, Birmingham, United Kingdom). Waist circumference was measured with a fiberglass tape at the narrowest point of the torso [17].

Assessment of exposures

Birth weight and length were recorded in the maternity hospitals at delivery. In the subsequent follow-ups the measurements were conducted at participant's home and at the research clinic in the 18-year old visit. On each occasion, weight and length or height were measured by trained field workers using standard equipment and protocols.

We studied growth patterns in several life periods: infancy (from birth to 1 year old), early and mid-childhood (1 to 4 and 4 to 11 years old, respectively), and early and late adolescence (11 to 15 and 15 to 18 years old, respectively). For each age interval, the separate effects of weight gain and linear growth were examined, using conditional relative weight and conditional length/height proposed by Adair et al [12]. Conditional relative weight takes into account current height and preceding weights and lengths or heights, and conditional length/height takes into account prior weight and length or height measures but not current weight [12].

To calculate these conditional measures, we computed sex-specific internally derived z-scores for weight and length or height at each follow-up. Then, we regressed the z-score size measurements (weight or height) at a given age, on z-scores at all previous measurements. The conditional measure is represented by the standardized residuals derived from the regression, and indicates the deviation from the individual's expected measures, in view of his or her previous

For peer review only - http://bmjopen.bmj.com/site/about/guidelines.xhtml

growth and the average growth of the cohort members. This could be interpreted as a measure of relatively faster or slower weight or length/height change over a period of time. For example, an adolescence with a positive value in CWh from 11 to 15 years, put on greater weight compared with his or her previous weights and length/heights and weights and length/heights of all cohort participants. As the conditional variables are uncorrelated, they can be included in a multiple regression model without breaking any assumption of collinearity [18].

Statistical analyses

We first described the outcomes by means (SD) (geometric means for asymmetric data: CRP and TGL). T-tests were used to estimate mean differences between sexes. To assess the association between each outcome and conditional growth we used linear regression and p-values were obtained by Wald's test. The outcomes were standardized to allow direct comparisons of the regression coefficients. We adjusted for following confounder factors: family income (in minimum wages), maternal education (completed years of schooling) at birth, self-reported skin color (white, black or others), breastfeeding (total duration of breastfeeding in months) and maternal smoking during pregnancy (yes/ no). Unadjusted and adjusted coefficients and statistical significance of associations did not differ markedly with the inclusion of confounders in our models, thus we presented only adjusted results. We included a term of interaction by sex in the model which provided statistical evidence of interaction in most of the associations, therefore, analyses are shown stratified by sex. Analyses were performed using Stata 12.1 (Stata Corp., College Station, Texas, and EUA

Results

For peer review only

At a mean age of 18.5 (SD 0.25) years 4106 adolescents were evaluated. Conditional relative weight and conditional length/height data across infancy, childhood and adolescence were available for 957 participants (23.4% of the 18-year old follow-up). Our main analysis samples consisted of those cohort members who had complete data on exposures, confounders and at least one outcome. Therefore our samples size were 917 for blood exams, and 946 for blood pressure, BMI and WC.

Mean levels of SBP were higher in boys than girls, as well as WC. On the other hand, girls had higher values on TC, HDL-C, LDL-C and TGL compared to boys. No differences were shown for DBP and BMI by sex (Table 1).

Table 1. Mean (SD) for outcomes at 18 years of age in the main analyses samples, stratified by sex. 1993 Pelotas Birth Cohort[a]

**Outcomes**	Boys N=438	Girls N=479	P-value
Protein-c (mg/L) [b]	0.64 (3.10)	1.35 (3.92)	<0.001
Total cholesterol (mg/dl)	151.31 (24.46)	172.29 (30.35)	<0.001
HDL cholesterol (mg/dl)	52.78 (8.75)	59.50 (10.54)	<0.001
LDL cholesterol (mg/dl)	83.55 (18.46)	97.14 (25.42)	<0.001
Triglycerides (mg/dl) [b]	70.81 (1.46)	74.79 (1.44)	0.03
Systolic blood pressure (mm/Hg) [c]	130.35 (11.56)	115.40 (10.04)	<0.001
Diastolic blood pressure (mm/Hg) [c]	70.54 (8.29)	69.71 (7.83)	0.12
Body mass index (kg/m^2) [c]	23.00 (4.25)	23.58 (5.05)	0.05
Waist circumference (cm) [c]	77.51 (9.71)	73.74 (10.32)	<0.001

Data are arithmetic mean (SD) unless otherwise indicated

[a] Main analyses sample includes individuals with complete data on all growth measures, all confounders and at least one outcome.

[b] Geometric mean (SD)

[c] Boys N= 447 ; girls N=499

For peer review only - http://bmjopen.bmj.com/site/about/guidelines.xhtml

Mean outcome values of the main analysis samples were compared with mean outcome values of all participants who had information on blood tests, blood pressure and anthropometric measures. Only few differences were observed between both samples. Boys included in the main analyses had lower CPR and higher HDL-C values as compared to boys who were evaluated at the 18-years follow-up. Among females, those included in the main analyses showed higher LDL-C than the total girls who were examined at the 18-years follow-up (Supplementary file 2). Proportions of males and females were slightly different in both samples, with males slightly sub-represented in the main analyses sample compared with the sample comprised by all participants who had information on blood tests, blood pressure and anthropometric measures (about 47.6% vs 49.8%). Comparing those included in the main analyses sample with those excluded, we observed statistical evidence of differences in mean of triglycerides and waist circumference among boys (Supplementary file 3).

Tables 2 and 3 show the association of size at birth, CWh and CH with cardiovascular risk markers at 18 years old.

CRP levels showed positive association with CWh during childhood and adolescence (from 1 to 4 years, 4 to 11 years and 11 to 15 years in both sexes, and 15 to 18 years only in girls). The association between CRP and weight gain appeared to be stronger and with an increasing trend across age periods in girls. Positive associations between CRP and CH between 1 and 4 years were found in both sexes (Tables 2 and 3).

Overall, lipid profile was associated with CWh gain during childhood and adolescence, especially in boys. CWh in the early childhood (1 to 4 years) was positive associated only with TGL, and CWh between 4 and 11 years was positively associated with LDL-C and TGL and negatively associated with HDL-C (Table 2). CWh during early and late adolescence (11 to 15

For peer review only

years and 15 to 18 years) were positively related with TC, LDL-C and TGL, and negatively related with HDL-C(Table 2). In girls, HDL-C was inversely associated only with CWh in mid-childhood (4 to 11 years) and both early and late adolescence (11 to 15 years and 15 to 18 years); whereas TC and LDL-C and TGL showed positive associations with CWh in late adolescence (Table 3).

Findings for SBP were similar for both sexes. However, DBP showed different patterns between sexes. Higher CWh during early and mid-childhood (1 to 4 years and 4 to 11 years) and through early and late adolescence (11 to15 years and 15 to 18 years) was associated with higher SBP in both sexes, and with higher DBP only in girls. Regarding linear growth, conditional length gain during the first year of life and CH gain during early and mid-childhood (1 to 4 years and 4 to 11 years) was related with increased levels of SBP in both sexes, whereas CH gain during early and mid-childhood were related with increased levels of DBP only in girls (Tables 2 and 3). Mean Arterial Pressure (MAP) was also assessed. In general, the results are consistent with those shown for SBP and DBP in both sexes (Supplementary file 4).

CWh in all age periods were associated with higher BMI and WC in boys and girls, with apparently larger coefficients after the first year of life (Table 2 and 3). CH between birth and age 1 year were positive related with BMI and WC only in boys. CH throughout early and mid-childhood (1 to 4 years and 4 to 11 years) was positive related with BMI and WC in both sexes (Table 2 and 3). CWh was more strongly related to BMI and WC than linear growth.

All the regression coefficients are shown in the original scales of outcomes (e.g.: mg/dl for lipid profile) in Supplementary file 5.

For peer review only

Table 2. Association of conditional relative weight and conditional height with cardiovascular risk markers at 18 years old in boys. 1993 Pelotas Birth Cohort.

	Cardiovascular risk markers								
	CRP	TC	HDL-C	LDL-C	TGL	SBP	DBP	BMI	WC
Conditional weight									
CWh 0 to1 y	-0.00 (-0.04; 0.04)	0.07 (-0.01; 0.14)	0.03 (-0.04; 0.10)	0.07 (-0.01; 0.15)	0.01 (-0.01; 0.03)	0.04 (-0.04; 0.112)	0.04 (-0.04; 0.13)	0.26 (0.18; 0.34)	0.27 (0.19; 0.35)
CWh 1 to 4 y	0.08 (0.03; 0.13)	0.03 (-0.05; 0.12)	-0.03 (-0.11; 0.05)	0.05 (-0.03; 0.11)	0.04 (0.01; 0.06)	0.09 (0.01; 0.17)	0.05 (-0.04; 0.15)	0.43 (0.35; 0.50)	0.40 (0.33; 0.48)
CWh 4 to11 y	0.10 (0.05; 0.14)	0.06 (-0.01; 0.13)	-0.08 (-0.15; -0.01)	0.09 (0.01; 0.16)	0.02 (0.01; 0.04)	0.08 (0.01; 0.16)	0.10 (0.01; 0.19)	0.48 (0.42; 0.53)	0.44 (0.38; 0.50)
CWh 11 to15 y	0.07 (0.02; 0.12)	0.13 (0.04; 0.20)	0.01 (-0.08; 0.09)	0.14 (0.07; 0.23)	0.04 (0.02; 0.06)	0.08 (0.01; 0.15)	0.08 (-0.01; 0.17)	0.33 (0.28; 0.38)	0.29 (0.24; 0.36)
CWh 15 to 18 y	0.04 (-0.01; 0.09)	0.16 (0.08; 0.23)	-0.11 (-0.19; -0.04)	0.18 (0.11; 0.26)	0.07 (0.05; 0.09)	0.12 (0.04; 0.20)	0.08 (-0.01; 0.18)	0.46 (0.44; 0.47)	0.47 (0.45; 0.50)
Conditional length/height									
CH 0 to 1 y	0.00 (-0.04; 0.05)	-0.01 (-0.09; 0.07)	-0.01 (-0.09; 0.07)	-0.00 (-0.08; 0.08)	-0.01 (-0.03; 0.02)	0.14 (0.06; 0.23)	0.08 (-0.02; 0.18)	0.11 (0.02; 0.19)	0.20 (0.11; 0.29)
CH 1 to 4 y	0.05 (0.01; 0.11)	0.06 (-0.02; 0.14)	0.03 (-0.05; 0.11)	0.05 (-0.04; 0.13)	0.02 (-0.01; 0.04)	0.09 (0.01; 0.17)	0.04 (-0.06; 0.14)	0.18 (0.09; 0.26)	0.24 (0.17; 0.33)
CH 4 to 11 y	0.00 (-0.05; 0.04)	0.06 (-0.03; 0.12)	-0.08 (-0.15; -0.01)	0.07 (-0.01; 0.15)	0.02 (-0.00; 0.05)	0.13 (0.06; 0.21)	0.21 (0.12; 0.30)	0.13 (0.07; 0.20)	0.18 (0.11; 0.26)
CH 11 to 15 y	-0.03 (-0.09; 0.01)	-0.00 (-0.08; 0.07)	0.01 (-0.07; 0.08)	0.00 (-0.08; 0.08)	-0.02 (-0.04; 0.00)	0.03 (-0.05; 0.11)	0.04 (-0.06; 0.14)	-0.02 (-0.08; 0.04)	0.02 (-0.04; 0.08)
CH 15 to 18 y	-0.00 (-0.05; 0.05)	-0.10 (-0.17; -0.02)	-0.03 (-0.11; 0.04)	-0.08 (-0.15; -0.00)	-0.02 (-0.04; 0.00)	0.07 (-0.01; 0.14)	0.02 (-0.08; 0.11)	-0.06 (-0.10; -0.01)	0.02 (-0.03; 0.08)

CRP: reactive-C protein, TC: total cholesterol, HDL-C: high-density lipoprotein cholesterol, LDL-C: low-density lipoprotein cholesterol, TGL: triglycerides, DBP: diastolic blood pressure, SBP: systolic blood pressure, BMI: body mass index, CC: waist circumference, CWh: conditional relative weight, CH: conditional height

Data are β (95% CI). The outcome variables were normalized. Regression coefficient (β) values were calculated with linear regression models and indicate the SD change in the outcome per SD change in the predictor. All models were adjusted for smoking during pregnancy, breastfeeding, mother's education (years of schooling) and household wealth (in minimum wages) at birth and skin color of the adolescent.

For peer review only

For peer review only - http://bmjopen.bmj.com/site/about/guidelines.xhtml

For peer review only

For peer review only - http://bmjopen.bmj.com/site/about/guidelines.xhtml

Table 3. Association of conditional relative weight and conditional height with cardiovascular risk markers at 18 years old in girls. 1993 Pelotas Birth Cohort

	Cardiovascular irsk markers								
	CRP	TC	HDL-C	LDL-C	TGL	SBP	DBP	BMI	WC
Conditional weight									
CWh 0 to 1 y	0.03 (-0.05; 0.12)	0.01 (-0.10; 0.09)	-0.04 (-0.13; 0.05)	-0.01 (-0.10; 0.10)	0.01 (-0.03; 0.06)	0.06 (-0.00; 0.13)	0.03 (-0.06; 0.12)	0.25 (0.15; 0.35)	0.22 (0.13; 0.31)
CWh 1 to 4 y	0.08 (-0.01; 0.18)	-0.03 (-0.13; 0.07)	-0.03 (-0.12; 0.08)	-0.04 (-0.15; 0.07)	0.02 (-0.03; 0.07)	0.12 (0.05; 0.18)	0.12 (0.03; 0.22)	0.56 (0.47; 0.65)	0.45 (0.37; 0.54)
CWh 4 to 11 y	0.11 (0.02; 0.20)	-0.04 (-0.13; 0.06)	-0.15 (-0.24; -0.07)	0.03 (-0.07; 0.13)	0.00 (-0.02; 0.02)	0.10 (0.04; 0.17)	0.13 (0.04; 0.22)	0.62 (0.55; 0.68)	0.53 (0.47; 0.59)
CWh 11 to 15 y	0.14 (0.06; 0.23)	0.00 (-0.09; 0.10)	-0.18 (-0.26; -0.09)	0.06 (-0.05; 0.15)	0.03 (0.01; 0.05)	0.15 (0.09; 0.21)	0.11 (0.03; 0.19)	0.45 (0.40; 0.50)	0.40 (0.34; 0.45)
CWh 15 to 18 y	0.22 (0.13; 0.31)	0.14 (0.04; 0.24)	-0.10 (-0.19; -0.02)	0.20 (0.09; 0.29)	0.03 (0.01; 0.06)	0.16 (0.10; 0.22)	0.13 (0.05; 0.22)	0.51 (0.50; 0.53)	0.47 (0.43; 0.49)
Conditional length/height									
CH 0 to 1 y	0.01 (-0.08; 0.12)	0.08 (-0.01; 0.17)	0.16 (0.08; 0.25)	0.03 (-0.07; 0.12)	-0.02 (-0.07; 0.02)	0.09 (0.02; 0.16)	0.01 (-0.08; 0.10)	0.01 (-0.08; 0.12)	0.08 (-0.00; 0.17)
CH 1 to 4 y	0.13 (0.04; 0.22)	0.06 (-0.04; 0.17)	0.11 (0.02; 0.20)	0.01 (-0.09; 0.12)	0.03 (-0.02; 0.08)	0.11 (0.04; 0.18)	0.11 (0.02; 0.21)	0.13 (0.02; 0.23)	0.22 (0.12; 0.31)
CH 4 to 11 y	-0.02 (-0.11; 0.07)	0.05 (-0.04; 0.15)	0.03 (-0.05; 0.12)	0.04 (-0.06; 0.14)	-0.00 (-0.02; 0.02)	0.08 (0.02; 0.14)	0.20 (0.11; 0.29)	0.12 (0.04; 0.20)	0.12 (0.04; 0.20)
CH 11 to 15 y	-0.01 (-0.10; 0.08)	-0.02 (-0.11; 0.08)	-0.07 (-0.15; 0.02)	0.02 (-0.09; 0.12)	-0.01 (-0.03; 0.02)	0.08 (0.02; 0.15)	0.02 (-0.07; 0.10)	-0.01 (-0.07; 0.06)	0.08 (0.01; 0.13)
CH 15 to 18 y	-0.10 (-0.19; -0.02)	0.00 (-0.09; 0.10)	-0.05 (-0.14; 0.03)	0.01 (-0.09; 0.11)	0.01 (-0.01; 0.03)	-0.00 (-0.07; 0.05)	0.00 (-0.08; 0.08)	-0.05 (-0.08; -0.03)	0.01 (-0.05; 0.05)

CRP: reactive-C protein, TC: total cholesterol, HDL-C: high-density lipoprotein cholesterol, LDL-C: low-density lipoprotein cholesterol, TGL: triglycerides, DBP: diastolic blood pressure, SBP: systolic blood pressure, BMI: body mass index, CC: waist circumference, CWh: conditional relative weight , CH : conditional height

Data are β (95% CI). The outcome variables were normalized. Regression coefficient (β) values were calculated with linear regression models and indicate the SD change in the outcome per SD change in the predictor. All models were adjusted for smoking during pregnancy, breastfeeding, mother's education (years of schooling), household wealth (in minimum wages) at birth and skin color of the adolescent.

For peer review only

Discussion

Our results showed some differences in the relative contributions of weight gain and linear growth to cardiovascular risk. In general, we observed that higher conditional relative weight in childhood (1 to 4 years and 4 to 11 years) was positively associated with CRP, LDL-C, TGL, SBP, DBP, BMI and WC in boys, and with CRP, DBP, SBP, BMI and WC among the girls (inverse associations with HDL-C in both sexes). Higher conditional relative weight in adolescence (11 to 15 years and 15 to 18 years) was positively associated with CRP, TC, LDL-C, TGL, SBP, BMI and WC and negatively associated with HDL-C in both sexes, and with DBP only in girls. Also, conditional relative weight during the first year of life was only related to BMI and WC at 18 years old adolescents of both sexes. By contrast, associations between linear growth and the cardiovascular markers showed a less consistent pattern when compared with weight gain. Greater conditional length at infancy was associated with higher values of SBP in boys and girls, and with BMI and WC only in boys.

Previous evidence from low-, middle- and high-income countries showed that excessive weight gain predicts subsequent metabolic and cardiovascular diseases when occurs in childhood, specifically after the second year of life. [12, 13, 19]. Overall, our findings showing association from early-childhood (after the first year of age) onwards are consistent with this evidence. The association between weight gain throughout life and CRP concentrations at young adulthood was examined in the 1982 Pelotas Birth Cohort. In agreement with our analysis, the study showed that excessive weight gain at all ages periods after the second year of life in both sexes were positively associated with CRP levels in 23 years old participants of both sexes [20]. We also found that excessive weight gain during childhood and adolescence was associated with increased total cholesterol, LDL-C and TGL and decreased HDL-C. Weight gain from birth to 4

For peer review only - http://bmjopen.bmj.com/site/about/guidelines.xhtml

years and lipid profile levels at 18 years were studied in the 1982 Cohort and negative associations were found between excessive weight gain from 2 to 4 years and HDL-C, although the association was reduced after controlling to current BMI [21]. In a study using data from the ALSPAC cohort in England, the authors observed that greater BMI in mid-childhood predicted higher blood pressure (SBP and DBP) in 17 years old adolescents [22].

Fall et al., assessed the relations between components of metabolic syndrome and weight gain at three periods from birth to age 28 years, age in which the outcomes were measured. In line with our findings, the study showed that greater weight gain in childhood (2 to 11 years) was associated with increased values of WC, SBP and TLG, while greater weight gain from 11 to 28 years predicted higher WC, TGL, total cholesterol and SBP and lower HDL-C. They also observed positive associations between rapid weight gain during the two first postnatal years and WC, SBP and TGL [19].

In concordance with analysis from the Vellore Birth Cohort in India [23], we observed that infancy (birth to 1 year) rapid conditional relative weight was positive associated with BMI and WC, but not with other cardiovascular markers. A meta-analyses assessing infant growth and subsequent obesity, also showed that infants who grew more rapidly had higher risk of developing obesity at posterior ages [7]. Studies that assessed the relation between growth and body composition showed that rapid weight gain in infancy were more related with fat free mass than fat mass in adulthood [12, 24]. In contrast, rapid weight gain from childhood onwards generally is associated with accumulation of greater fat mass compared with free fat mass [11, 12, 24-26]. These findings may explain the positive associations of relative weight gain at all age periods with BMI, as this indicator does not distinguish body fat from free fat mass.

For peer review only - http://bmjopen.bmj.com/site/about/guidelines.xhtml

In relation to linear growth, we observed that faster conditional height mainly in childhood was found to be positive associated with blood pressure (SBP and DBP), BMI and WC at 18 years old in both sexes.A previous report with data of the same cohort examined the association between conditional growth at three different age ranges up to 4 years old, BMI and blood pressure at 15 years, and found positive associations between conditional height from 1 to 4 years and both outcomes, although the association with SBP became insignificant adjusted for BMI at 15 years [27]. Haugaard et al, found positive association between linear growth during childhood and SBP and WC at age 8 years old [28]. The previously mentioned Vellore Birth Cohort showed that rapid conditional height throughout life course was positive associated with blood pressure and WC in young adults [23]. It is known that blood pressure is higher in taller people, this may be result of an adaptation of the vascular function to perfuse a longer arterial tree. However, inverse relations between adult height and cardiovascular diseases have been described as well [29], which suggests that this adaption possibly has not pathological consequences.

Strengths of this study include a population based sample and the availability of several anthropometric and biological markers of cardiovascular risk. Furthermore, our data have been collected prospectively since birth by trained staff with the use of standardized methods, reducing the susceptibility to misclassification. We also highlight the use of conditional growth to examine highly correlated measurements typical of longitudinal studies, and the assessment of the separate contributions of linear growth and weight gain relative to linear growth. Nevertheless, the assessment of relative weight gain does not distinguish between free-fat and fat mass gain.

For peer review only - http://bmjopen.bmj.com/site/about/guidelines.xhtml

We acknowledge some limitations of our study. First, our analysis were carried out only by those cohort members with anthropometric data from several follow-ups, including sub-samples. The potential impact of losses to follow-up on our results is difficult to assess. Second, we cannot rule out the possibility of random significant associations, as it is known that multiple hypothesis testing lead to increased Type I error. Third, measurements at age 2 years old were not available in the 1993 Pelotas Birth Cohort, and therefore we had no ability to assess growth at this age point. The inclusion of age 2 years old in longitudinal studies assessing the associations between growth and cardiovascular risk is very important due to growing evidence showing that excessive relative weight gain denotes increased risk for cardiovascular health when occurred after the age of 2 years. Even though, we do not have evidence of the impact of conditional growth at age 2 on later outcomes, our findings for the first year of life add to the evidence that improving nutrition during the "first days of life" would result on long-term benefits on health, as greater infant weight gain and linear growth during this period have more benefits than risks for health, specially improvement of capital human outcomes as schooling and final achieved height [12, 13].

Although we studied an particular population (e.g.: children born in 1993 in the urban area of Pelotas, Brazil), we observed many similarities of results with several studies, revealing that our findings may be generalisable to populations in other low- and middle-income countries.

Formatted: Font color: Text 1

Given that cardiovascular risk can track from adolescence to adulthood [30, 31], a better understanding of the possible adverse effects of growth patterns at earlier stages in life can help to develop interventions aimed at preventing subsequent chronic diseases. Based on this study and other published evidence, we conclude that excessive weight gain from childhood onward may have an adverse effect on cardiovascular health later in life. This reinforces efforts to inform

For peer review only - http://bmjopen.bmj.com/site/about/guidelines.xhtml

strategies to avoid putting on weight children after their 2 first years of life for cardiovascular prevention. Evidence regarding linear growth throughout birth to late adolescence and subsequent cardiovascular risk remains unclear and needs further investigation.

Contributorship: RB, MCFA and MCR-M designed the study. RB performed the analysis. RB, MCR-M, MCFA and VMS contributed to the interpretation of the results and critical revision of the manuscript. HDG, AMM, IOO, MCFA participated in the design and conduct of the original cohort studies as well as in interpreting results and reviewing the manuscript. RB wrote the manuscript, and all authors contributed to and approved the final version.

Competing interests: None declared

Funding: This article is based on data from the study "Pelotas Birth Cohort, 1993" conducted by Postgraduate Program in Epidemiology at Universidade Federal de Pelotas with the collaboration of the Brazilian Public Health Association (ABRASCO). From 2004 to 2013, the Wellcome Trust supported the 1993 birth cohort study. The European Union, National Support Program for Centers of Excellence (PRONEX), the Brazilian National Research Council (CNPq), and the Brazilian Ministry of Health supported previous phases of the study.

Ethical approval: The study and its protocols were approved by the School of Medicine Ethics Committee of the Federal University of Pelotas. All participants or their legal representatives voluntarily signed a consent letter (verbal consent was provided in perinatal phase) prior to participation in each follow-up.

Acknowledgements: We are grateful to all the adolescents who took part in the Pelotas birth cohorts, and the Pelotas teams, including research scientists, interviewers, workers and volunteers.

Data sharing: Due to confidentiality restrictions related to the ethics approval for this study, no identifying information about participants may be released. As recipients, the authors were allowed to publish analytic results from the data, but not the data itself, due to confidentiality conditions.

For peer review only

References

1. WHO. Global Health Risks: mortality and burden of disease atrributable to selected major risks. 2009.
2. Wells JC. The programming effects of early growth. Early human development. 2007;83(12):743-8. Epub 2007/10/02.
3. Whincup PH, Kaye SJ, Owen CG, Huxley R, Cook DG, Anazawa S, et al. Birth weight and risk of type 2 diabetes: a systematic review. Jama. 2008;300(24):2886-97. Epub 2008/12/26.
4. Newsome CA, Shiell AW, Fall CH, Phillips DI, Shier R, Law CM. Is birth weight related to later glucose and insulin metabolism?--A systematic review. Diabetic medicine : a journal of the British Diabetic Association. 2003;20(5):339-48. Epub 2003/05/20.
5. Eriksson JG. Early growth and coronary heart disease and type 2 diabetes: findings from the Helsinki Birth Cohort Study (HBCS). The American journal of clinical nutrition. 2011;94(6 Suppl):1799S-802S. Epub 2011/05/27.
6. Barker DJ. Fetal programming of coronary heart disease. Trends in endocrinology and metabolism: TEM. 2002;13(9):364-8. Epub 2002/10/09.
7. Baird J, Fisher D, Lucas P, Kleijnen J, Roberts H, Law C. Being big or growing fast: systematic review of size and growth in infancy and later obesity. BMJ (Clinical research ed). 2005;331(7522):929. Epub 2005/10/18.
8. Fisher D, Baird J, Payne L, Lucas P, Kleijnen J, Roberts H, et al. Are infant size and growth related to burden of disease in adulthood? A systematic review of literature. International journal of epidemiology. 2006;35(5):1196-210. Epub 2006/07/18.
9. Monteiro PO, Victora CG. Rapid growth in infancy and childhood and obesity in later life--a systematic review. Obesity reviews : an official journal of the International Association for the Study of Obesity. 2005;6(2):143-54. Epub 2005/04/20.
10. Ong KK, Ahmed ML, Emmett PM, Preece MA, Dunger DB. Association between postnatal catch-up growth and obesity in childhood: prospective cohort study. BMJ (Clinical research ed). 2000;320(7240):967-71. Epub 2001/02/07.
11. Reilly JJ, Armstrong J, Dorosty AR, Emmett PM, Ness A, Rogers I, et al. Early life risk factors for obesity in childhood: cohort study. BMJ (Clinical research ed). 2005;330(7504):1357. Epub 2005/05/24.
12. Adair LS, Fall CH, Osmond C, Stein AD, Martorell R, Ramirez-Zea M, et al. Associations of linear growth and relative weight gain during early life with adult health and human capital in countries of low

For peer review only

For peer review only - http://bmjopen.bmj.com/site/about/guidelines.xhtml

and middle income: findings from five birth cohort studies. Lancet. 2013;382(9891):525-34. Epub 2013/04/02.
13. Victora CG, Adair L, Fall C, Hallal PC, Martorell R, Richter L, et al. Maternal and child undernutrition: consequences for adult health and human capital. Lancet. 2008;371(9609):340-57. Epub 2008/01/22.
14. Victora CG, Hallal PC, Araujo CL, Menezes AM, Wells JC, Barros FC. Cohort profile: the 1993 Pelotas (Brazil) birth cohort study. International journal of epidemiology. 2008;37(4):704-9. Epub 2007/09/12.
15. Victora CG, Araújo CLP, Menezes AMB, Hallal PC, Vieira MdF, Neutzling MB, et al. Methodological aspects of the 1993 Pelotas (Brazil) birth cohort study. Revista de saude publica. 2006;40(1):39-46.
16. Goncalves H, Assuncao MC, Wehrmeister FC, Oliveira IO, Barros FC, Victora CG, et al. Cohort profile update: The 1993 Pelotas (Brazil) birth cohort follow-up visits in adolescence. International journal of epidemiology. 2014;43(4):1082-8. Epub 2014/04/15.
17. Lohman TG, Roche AF, Martorell R. Anthropometric standardization reference manual: Human kinetics books; 1988.
18. Keijzer-Veen MG, Euser AM, van Montfoort N, Dekker FW, Vandenbroucke JP, Van Houwelingen HC. A regression model with unexplained residuals was preferred in the analysis of the fetal origins of adult diseases hypothesis. J Clin Epidemiol. 2005;58(12):1320-4. Epub 2005/11/18.
19. Fall CH, Sachdev HS, Osmond C, Lakshmy R, Biswas SD, Prabhakaran D, et al. Adult metabolic syndrome and impaired glucose tolerance are associated with different patterns of BMI gain during infancy: Data from the New Delhi Birth Cohort. Diabetes Care. 2008;31(12):2349-56. Epub 2008/10/07.
20. Nazmi A, Gonzalez DC, Oliveira IO, Horta BL, Gigante DP, Victora CG. Life course weight gain and C-reactive protein levels in young adults: findings from a Brazilian birth cohort. American journal of human biology : the official journal of the Human Biology Council. 2009;21(2):192-9. Epub 2008/12/25.
21. Horta BL, Victora CG, Lima RC, Post P. Weight gain in childhood and blood lipids in adolescence. Acta paediatrica (Oslo, Norway : 1992). 2009;98(6):1024-8. Epub 2009/06/02.
22. Howe LD, Chaturvedi N, Lawlor DA, Ferreira DL, Fraser A, Davey Smith G, et al. Rapid increases in infant adiposity and overweight/obesity in childhood are associated with higher central and brachial blood pressure in early adulthood. J Hypertens. 2014;32(9):1789-96. Epub 2014/07/16.
23. Antonisamy B, Vasan SK, Geethanjali FS, Gowri M, Hepsy YS, Richard J, et al. Weight Gain and Height Growth during Infancy, Childhood, and Adolescence as Predictors of Adult Cardiovascular Risk. J Pediatr. 2016. Epub 2016/11/09.
24. Victora CG, Sibbritt D, Horta BL, Lima RC, Cole T, Wells J. Weight gain in childhood and body composition at 18 years of age in Brazilian males. Acta paediatrica (Oslo, Norway : 1992). 2007;96(2):296-300. Epub 2007/04/13.
25. Wells JC, Hallal PC, Wright A, Singhal A, Victora CG. Fetal, infant and childhood growth: relationships with body composition in Brazilian boys aged 9 years. International journal of obesity (2005). 2005;29(10):1192-8. Epub 2005/08/17.
26. Sachdev HS, Fall CH, Osmond C, Lakshmy R, Dey Biswas SK, Leary SD, et al. Anthropometric indicators of body composition in young adults: relation to size at birth and serial measurements of body mass index in childhood in the New Delhi birth cohort. The American journal of clinical nutrition. 2005;82(2):456-66. Epub 2005/08/10.
27. Menezes AM, Hallal PC, Dumith SC, Matijasevich AM, Araujo CL, Yudkin J, et al. Adolescent blood pressure, body mass index and skin folds: sorting out the effects of early weight and length gains. Journal of epidemiology and community health. 2012;66(2):149-54. Epub 2011/02/18.

For peer review only - http://bmjopen.bmj.com/site/about/guidelines.xhtml

28. Haugaard LK, Baker JL, Perng W, Belfort MB, Rifas-Shiman SL, Switkowski K, et al. Growth in Total Height and Its Components and Cardiometabolic Health in Childhood. Plos One. 2016;11(9):e0163564. Epub 2016/09/23.
29. Wannamethee SG, Shaper AG, Whincup PH, Walker M. Adult height, stroke, and coronary heart disease. Am J Epidemiol. 1998;148(11):1069-76. Epub 1998/12/16.
30. Camhi SM, Katzmarzyk PT. Tracking of cardiometabolic risk factor clustering from childhood to adulthood. Pediatric Obesity. 2010;5(2):122-9.
31. Harding S, Silva MJ, Molaodi OR, Enayat ZE, Cassidy A, Karamanos A, et al. Longitudinal study of cardiometabolic risk from early adolescence to early adulthood in an ethnically diverse cohort. BMJ open. 2016;6(12):e013221.

For peer review only

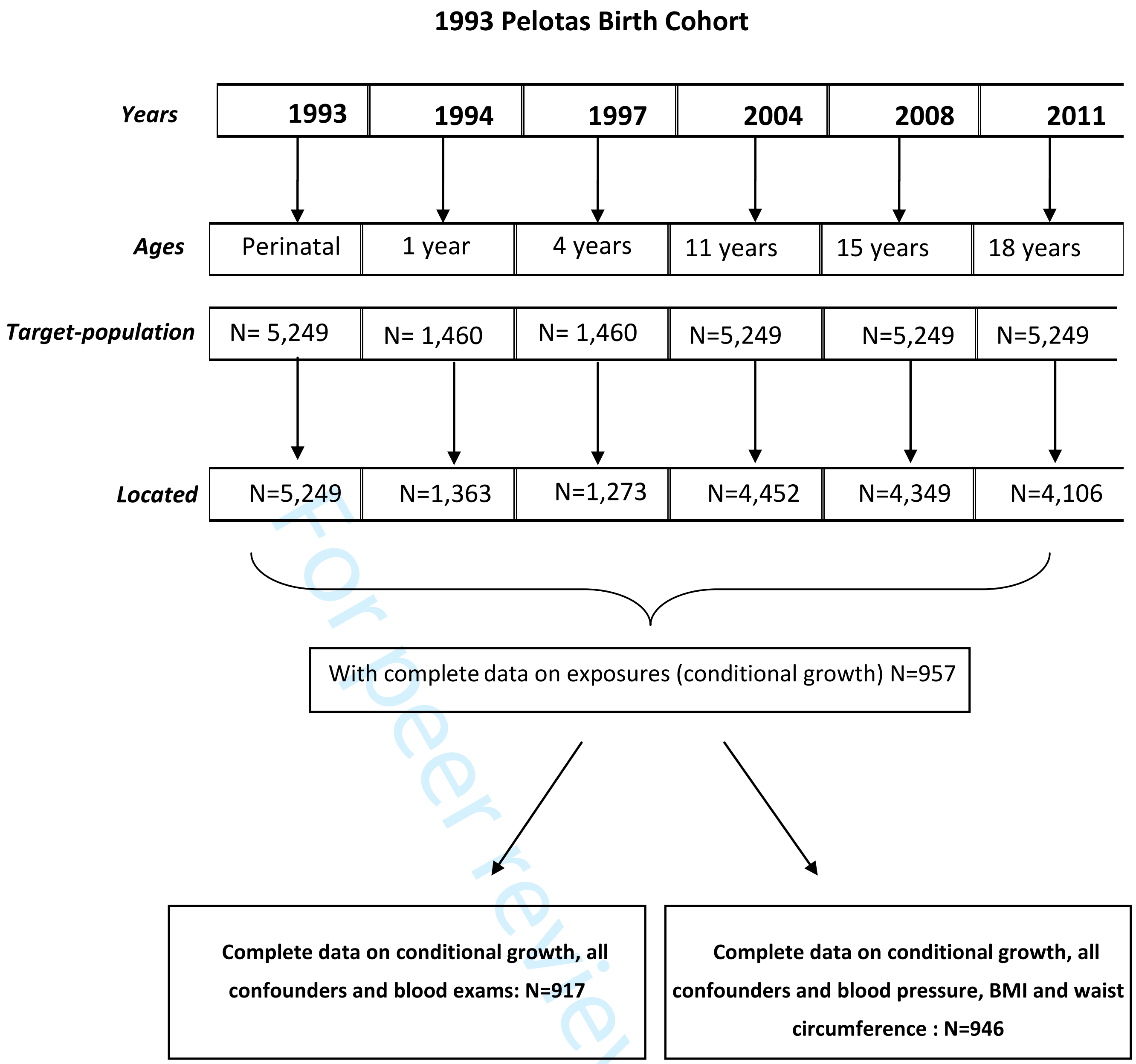

Figure 1. Description of the 1993 Pelotas (Brazil) Birth Cohort.

For peer review only

Supplementary file 2

Table 1. Mean (SD) for outcomes at 18 years of age in the main analyses samples* and samples comprised by all the adolescents with outcomes measures at the 18 years follow-up, stratified by sex. 1993 Pelotas Birth Cohort.

	Boys					Girls				
**Outcomes**	N	Main analyses sample	N	All participants	p-value*	N	Main analyses sample	N	All participants	p-value*
Plasma glucose (mg/dl)	438	93.60 (20.88)	1933	93.92 (22.65)	0.79	479	88.41 (15.36)	1936	89.74 (18.64)	0.15
HbA1c (%)	438	4.97 (0.60)	1924	4.96 (0.57)	0.74	475	4.86 (0.50)	1910	4.84 (0.52)	0.45
C-reactive Protein (mg/L)	438	0.64 (3.10)	1933	0.67 (3.26)	<0.01	479	1.35 (3.92)	1936	1.35 (3.86)	0.88
Total cholesterol (mg/dl)	438	151.31 (24.46)	1933	152.72 (24.55)	0.27	479	172.29 (30.35)	1936	169.80 (29.19)	0.10
HDL cholesterol (mg/dl)	438	52.78 (8.75)	1933	51.78 (8.75)	0.03	479	59.50 (10.54)	1936	59.84 (10.92)	0.54
LDL cholesterol (mg/dl)	438	83.55 (18.46)	1933	84.28 (20.23)	0.49	479	97.14 (25.42)	1936	93.89 (23.84)	0.01
Triglycerides (mg/dl)	438	70.81 (1.46)	1933	73.56 (1.51)	0.16	479	74.79 (1.44)	1936	74.93 (1.49)	0.62
Systolic blood pressure (mm/Hg)	447	130.3 (11.56)	1979	130.71 (11.90)	0.52	499	115.40 (10.04)	2008	115.06 (9.95)	0.50
Diastolic blood pressure (mm/Hg)	447	70.54 (8.29)	1979	70.95 (7.94)	0.33	499	69.64 (7.83)	2008	69.46 (7.75)	0.64
Body mass index	447	22.99 (4.24)	1970	23.36 (4.23)	0.10	499	23.58 (5.06)	2003	23.52 (4.76)	0.80
Waist circumference (cm)	447	77.51 (9.71)	1972	78.45 (9.61)	0.06	499	73.74 (10.31)	2005	73.75 (9.75)	0.98

Data are arithmetic mean (SD) unless otherwise indicated
* Geometric mean (SD)
*Main analyses samples includes individuals with complete data on all growth measures, all confounders and at least one outcome.
*p-value for T-test. C-reactive protein and triglycerides were log transformed to performed de test.

For peer review only

For peer review only

Supplementary file 3

Table 1. Differences in mean (SD) for outcomes at 18 years of age between included and excluded participants, stratified by sex. 1993 Pelotas Birth Cohort.

Outcomes	Boys					Girls				
	N	Main analyses sample	N	Excluded	p-value*	N	Main analyses sample	N	Excluded	p-value*
C-reactive Protein (mg/L)	438	0.64 (3.10)	1495	0.68 (3.30)	0.26	479	1.35 (3.92)	1457	1.35 (3.83)	0.95
Total cholesterol (mg/dl)	438	151.31 (24.46)	1495	153.13 (25.11)	0.17	479	172.29 (30.35)	1457	168.9 (28.76)	0.06
HDL cholesterol (mg/dl)	438	52.78 (8.75)	1495	51.70 (8.75)	0.44	479	59.50 (10.54)	1457	59.95 (11.04)	0.43
LDL cholesterol (mg/dl)	438	83.55 (18.46)	1495	84.48 (20.73)	0.40	479	97.14 (25.42)	1457	92.82 (23.20)	<0.00
Triglycerides (mg/dl)	438	70.81 (1.46)	1495	74.38 (1.52)	0.03	479	74.79 (1.44)	1457	74.97 (1.50)	0.91
Systolic blood pressure (mm/Hg)	447	130.30 (11.56)	1532	130.83 (12.00)	0.40	499	115.40 (10.04)	1509	114.99 (9.94)	0.57
Diastolic blood pressure (mm/Hg)	447	70.54 (8.29)	1532	71.09 (7.83)	0.13	499	69.64 (7.83)	1509	69.40 (7.72)	0.56
Body mass index (kg/m^2)	447	22.99 (22.60)	1523	23.47 (23.26)	0.08	499	23.58 (23.14)	1504	23.50 (23.27)	0.75
Waist circumference (cm)	447	77.51 (9.71)	1525	78.72 (78.24)	0.03	499	73.74 (10.31)	1506	73.76 (73.27)	0.97

Data are arithmetic mean (SD) unless otherwise indicated
* Geometric mean (SD)
*Included participants (main analyses samples): includes individuals with complete data on all growth measures, all confounders and at least one outcome.
*p-value for T-test. C-reactive protein and triglycerides were log transformed to performed de test.

For peer review only

For peer review only

For peer review only - http://bmjopen.bmj.com/site/about/guidelines.xhtml

Supplementary file 4

Table 1. Association of conditional relative weight and conditional height with mean arterial pressure at 18 years old in boys and girls. 1993 Pelotas Birth Cohort.

	Mean arterial pressure (MAP)	
	Boys	Girls
Conditional weight		
CWh 0 to1 y	0.04 (-0.04; 0.13)	0.06 (-0.03; 0.14)
CWh 1 to 4 y	0.08 (-0.01; 0.17)	0.14 (0.05; 0.22)
CWh 4 to11 y	0.11 (0.02; 0.19)	0.14 (0.06; 0.22)
CWh 11 to15 y	0.08 (0.01; 0.18)	0.15 (0.07; 0.22)
CWh 15 to 18 y	0.11 (0.02; 0.20)	0.16 (0.09; 0.24)
Conditional length/height		
CH 0 to 1 y	0.12 (0.31; 0.22)	0.05 (-0.03; 0.13)
CH 1 to 4 y	0.07 (-0.20; 0.17)	0.13 (0.04; 0.22)
CH 4 to 11 y	0.20 (0.11; 0.28)	0.17 (0.09; 0.24)
CH 11 to 15 y	0.04 (-0.05; 0.13)	0.05 (-0.02; 0.13)
CH 15 to 18 y	0.05 (-0.04; 0.13)	0.00 (-0.07; 0.07)

CWh: conditional relative weight, CH: conditional height

Data are β (95% CI). The outcome variables were normalized. Regression coefficient (β) values were calculated with linear regression models and indicate the SD change in the outcome per SD change in the predictor. All models were adjusted for mother's education (years of schooling) and household wealth (in minimum wages) at birth and skin color of the adolescent.

For peer review only

Supplementary file 5

Table 1. Association of weight and length at birth, conditional relative weight and conditional height with cardiovascular risk markers at 18 years old in boys. 1993 Pelotas Birth Cohort.

	Cardiovascular risk markers								
	CRP (mg/L)	TC (mg/dl)	HDL-C (mg/dl)	LDL-C (mg/dl)	TGL (mg/dl)	SBP (mm/Hg)	DBP (mm/Hg)	BMI (kg/m^2)	WC (cm)
Conditional relative weight									
CWh 0 to1 y	0.99	2.41	0.37	1.74	1.01	0.60	0.40	1.19	2.77
CWh 1 to 4 y	1.27	0.83	-0.41	0.85	1.05	1.15	0.39	1.92	4.03
CWh 4 to 11 y	1.24	1.58	-0.84	1.96	1.02	1.17	0.78	2.17	4.37
CWh 11to 15 y	1.14	3.50	0.03	3.10	1.05	0.97	0.60	1.50	2.98
CWh 15 to 18 y	1.18	4.48	-1.16	3.19	1.12	1.00	0.68	2.08	4.73
Conditional length/height									
CH 0 to 1 y	0.99	-0.13	-0.06	0.16	0.99	1.97	0.66	0.49	2.04
CH 1 to 4 y	1.14	1.74	0.29	1.04	1.05	1.25	0.29	0.85	2.43
CH 4 to 11 y	1.05	1.32	-0.88	1.69	1.03	1.80	1.66	0.60	1.84
CH 11 to 15 y	0.96	-0.21	0.33	-0.06	0.97	0.45	0.30	-0.09	0.18
CH 15 to 18 y	0.97	-2.75	-0.36	-1.76	0.97	0.90	0.13	-0.25	0.23

TC: total cholesterol, HDL-C: high-density lipoprotein cholesterol, LDL-C: low-density lipoprotein cholesterol, TGL: triglycerides, DBP: diastolic blood pressure, SBP: systolic blood pressure, BMI: body mass index, CC: waist circumference, CWh: conditional relative weight gain, CH : conditional height gain

Data are β (95% CI). The outcome variables are presented in original scales. Regression coefficient (β) values were calculated with linear regression models and indicate a unit change in the outcome (e.g.: 1 cm or 1 mm/Hg) per SD change in the predictor. All models were adjusted for mother's education (years of schooling) and household wealth (in minimum wages) at birth and skin color of the adolescent. The models for plasma glucose concentrations were further adjusted for time from previous meal.

For peer review only

Table 2. Association of Conditional relative weight and conditional height with cardiovascular risk markers at 18 years old in girls. 1993 Pelotas Birth Cohort

	Cardiovascular risk markers								
	CRP (mg/L)	TC (mg/dl)	HDL-C (mg/dl)	LDL-C (mg/dl)	TGL (mg/dl)	SBP (mm/Hg)	DBP (mm/Hg)	BMI (kg/m^2)	WC (cm)
Conditional relative weight									
CWh 0 to1 y	1.07	0.21	-0.11	-0.40	1.01	0.96	0.26	1.15	2.31
CWh 1 to 4 y	1.24	-1.10	-0.33	-0.98	1.02	1.56	0.96	2.53	4.48
CWh 4 to 11 y	1.21	-1.05	-1.63	0.69	1.00	1.42	1.10	2.77	5.28
CWh 11to 15 y	1.24	0.27	-1.91	1.22	1.05	2.00	0.87	2.02	3.95
CWh 15 to 18 y	1.59	3.88	-1.13	4.49	1.06	2.19	1.02	2.32	4.63
Conditional length/height									
CH 0 to 1 y	0.99	2.36	1.76	0.70	0.99	1.21	0.06	0.10	0.94
CH 1 to 4 y	1.16	1.89	1.04	0.33	1.02	1.53	0.91	0.57	2.20
CH 4 to 11 y	1.01	1.30	0.37	0.90	0.99	1.12	1.65	0.52	1.20
CH 11 to 15 y	0.97	-0.56	-0.75	0.29	0.99	1.12	0.16	-0.03	0.68
CH 15 to 18 y	0.85	0.16	-0.53	0.16	1.02	-0.11	-0.01	-0.23	0.01

HbA1c: glycated hemoglobin, CRP: reactive-C protein, TC: total cholesterol, HDL-C: high-density lipoprotein cholesterol, LDL-C: low-density lipoprotein cholesterol, TGL: triglycerides, DBP: diastolic blood pressure, SBP: systolic blood pressure, BMI: body mass index, CC: waist circumference, CWh: conditional relative weight gain, CH : conditional height gain

Data are β (95% CI). The outcome variables are presented in original scales. Regression coefficient (β) values were calculated with linear regression models and indicate a unit change in the outcome (e.g.: 1 cm or 1 mm/Hg) per SD change in the predictor. All models were adjusted for mother's education (years of schooling) and household wealth (in minimum wages) at birth and skin color of the adolescent. The models for plasma glucose concentrations were further adjusted for time from previous meal.

For peer review only

STROBE Statement—checklist of items that should be included in reports of observational studies

	Item No	Recommendation	Pag.
**Title and abstract**	1	(*a*) Indicate the study's design with a commonly used term in the title or the abstract	1
		(*b*) Provide in the abstract an informative and balanced summary of what was done and what was found	1
**Introduction**			
Background/rationale	2	Explain the scientific background and rationale for the investigation being reported	4
Objectives	3	State specific objectives, including any prespecified hypotheses	4
**Methods**			
Study design	4	Present key elements of study design early in the paper	5
Setting	5	Describe the setting, locations, and relevant dates, including periods of recruitment, exposure, follow-up, and data collection	5
Participants	6	(*a*) *Cohort study*—Give the eligibility criteria, and the sources and methods of selection of participants. Describe methods of follow-up<br>*Case-control study*—Give the eligibility criteria, and the sources and methods of case ascertainment and control selection. Give the rationale for the choice of cases and controls<br>*Cross-sectional study*—Give the eligibility criteria, and the sources and methods of selection of participants	5
		(*b*) *Cohort study*—For matched studies, give matching criteria and number of exposed and unexposed<br>*Case-control study*—For matched studies, give matching criteria and the number of controls per case	NA
Variables	7	Clearly define all outcomes, exposures, predictors, potential confounders, and effect modifiers. Give diagnostic criteria, if applicable	6-7
Data sources/ measurement	8*	For each variable of interest, give sources of data and details of methods of assessment (measurement). Describe comparability of assessment methods if there is more than one group	6-7
Bias	9	Describe any efforts to address potential sources of bias	NA
Study size	10	Explain how the study size was arrived at	8
Quantitative variables	11	Explain how quantitative variables were handled in the analyses. If applicable, describe which groupings were chosen and why	8
Statistical methods	12	(*a*) Describe all statistical methods, including those used to control for confounding	8
		(*b*) Describe any methods used to examine subgroups and interactions	NA
		(*c*) Explain how missing data were addressed	8
		(*d*) *Cohort study*—If applicable, explain how loss to follow-up was addressed<br>*Case-control study*—If applicable, explain how matching of cases and controls was addressed<br>*Cross-sectional study*—If applicable, describe analytical methods taking account of sampling strategy	8
		(*e*) Describe any sensitivity analyses	

For peer review only

Continued on next page

For peer review only

For peer review only - http://bmjopen.bmj.com/site/about/guidelines.xhtml

**Results**			
Participants	13*	(a) Report numbers of individuals at each stage of study—eg numbers potentially eligible, examined for eligibility, confirmed eligible, included in the study, completing follow-up, and analysed	8
		(b) Give reasons for non-participation at each stage	NA
		(c) Consider use of a flow diagram	NA
Descriptive data	14*	(a) Give characteristics of study participants (eg demographic, clinical, social) and information on exposures and potential confounders	9
		(b) Indicate number of participants with missing data for each variable of interest	8
		(c) *Cohort study*—Summarise follow-up time (eg, average and total amount)	
Outcome data	15*	*Cohort study*—Report numbers of outcome events or summary measures over time	Table 1
		Case-control study—Report numbers in each exposure category, or summary measures of exposure	NA
		Cross-sectional study—Report numbers of outcome events or summary measures	NA
Main results	16	(*a*) Give unadjusted estimates and, if applicable, confounder-adjusted estimates and their precision (eg, 95% confidence interval). Make clear which confounders were adjusted for and why they were included	Table 2 and 3
		(*b*) Report category boundaries when continuous variables were categorized	NA
		(*c*) If relevant, consider translating estimates of relative risk into absolute risk for a meaningful time period	NA
Other analyses	17	Report other analyses done—eg analyses of subgroups and interactions, and sensitivity analyses	
**Discussion**			
Key results	18	Summarise key results with reference to study objectives	14
Limitations	19	Discuss limitations of the study, taking into account sources of potential bias or imprecision. Discuss both direction and magnitude of any potential bias	16
Interpretation	20	Give a cautious overall interpretation of results considering objectives, limitations, multiplicity of analyses, results from similar studies, and other relevant evidence	14-17
Generalisability	21	Discuss the generalisability (external validity) of the study results	16
**Other information**			
Funding	22	Give the source of funding and the role of the funders for the present study and, if applicable, for the original study on which the present article is based	18

*Give information separately for cases and controls in case-control studies and, if applicable, for exposed and unexposed groups in cohort and cross-sectional studies.

Note: An Explanation and Elaboration article discusses each checklist item and gives methodological background and published examples of transparent reporting. The STROBE checklist is best used in conjunction with this article (freely available on the Web sites of PLoS Medicine at http://www.plosmedicine.org/, Annals of Internal Medicine at http://www.annals.org/, and Epidemiology at http://www.epidem.com/). Information on the STROBE Initiative is available at www.strobe-statement.org.

For peer review only

For peer review only - http://bmjopen.bmj.com/site/about/guidelines.xhtml
